# Review of the odd chrysidid genus *Loboscelidia* Westwood, 1874 (Hymenoptera, Chrysididae, Loboscelidiinae)

**DOI:** 10.3897/zookeys.213.2985

**Published:** 2012-08-01

**Authors:** Lynn S. Kimsey

**Affiliations:** 1 Bohart Museum of Entomology, Department of Entomology, University of California, Davis, California, 95616, USA

**Keywords:** Viet Nam, Borneo, Thailand, India, Sri Lanka, Philippines, Australia

## Abstract

The chrysidid genus *Loboscelidia* is reviewed and 11 new species are described, including *Loboscelidia cinnamonea* (Borneo), *Loboscelidia fulgens* (Viet Nam), *Loboscelidia fulva* (Thailand), *Loboscelidia incompleta* (India), *Loboscelidia kafae* (Borneo), *Loboscelidia laminata* (Viet Nam), *Loboscelidia meifungae* (Borneo), *Loboscelidia nasiformis* (Thailand), *Loboscelidia nitidula* (Thailand), *Loboscelidia pecki* (Viet Nam), and *Loboscelidia sisik* (Borneo). A key to males of the species of *Loboscelidia* is given.

## Introduction

Loboscelidiinae is one of the smaller subfamilies in the family Chrysididae. The subfamily contains two genera, *Loboscelidia* Westwood, 1874 and *Rhadinoscelidia* Kimsey, 1988. As of the publication of Kimsey and Bohart (1991), *Loboscelidia* contained 30 species and *Rhadinoscelidia* one species. Since then four *Loboscelidia* and two *Rhadinoscelidia* species have been added (Kojima and Ubaidillah 2003, Terayama et al. 1998, Xu et al. 2006). An additional 11 new *Loboscelidia* species are described below. This study focuses on males and their characteristics as the systematics of the group is focused primarily on this sex due to the rareness of females in collections and the strong sexual dimorphism between males and females.

The subfamily is primarily south Asian with four northern Australian species. Every major south Asian island may have at least one endemic species of *Loboscelidia*, and every new intensive collecting effort using Malaise traps or flight-intercept traps turns up new species. Thus, the loboscelidiine fauna appears to be largely under-sampled.

**Figure 1.**
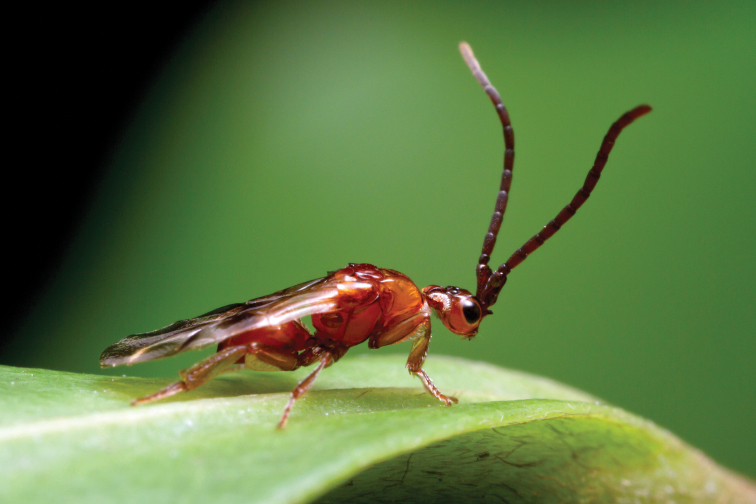
Habitus photograph of male *Loboscelidia* sp. in Queensland, Australia. Photo courtesy of Alex Wild; myrmecos.net.

Loboscelidiines are among the most aberrant-looking and highly modified chrysidids, and as a result their actual family and even superfamily placement has varied considerably over the years. These are small-bodied, non-metallic brown wasps, with a superficial resemblance to members of the family Diapriidae (see Fig. 1). In fact Westwood (1874) originally described *Loboscelidia* as a species of diapriid (Superfamily Proctotrupoidea). Ashmead (1902) then moved the genus to the family Figitidae (Superfamily Cynipoidea). Maa and Yoshimoto (1961) then moved the genus *Loboscelidia* into its own family, Loboscelidiidae (Superfamily Bethyloidea). Finally, after making a detailed analysis of the metasomal morphology Day (1978) concluded that the group actually belonged in the family Chrysididae (Superfamily Chrysidoidea).

**Figure 2.**
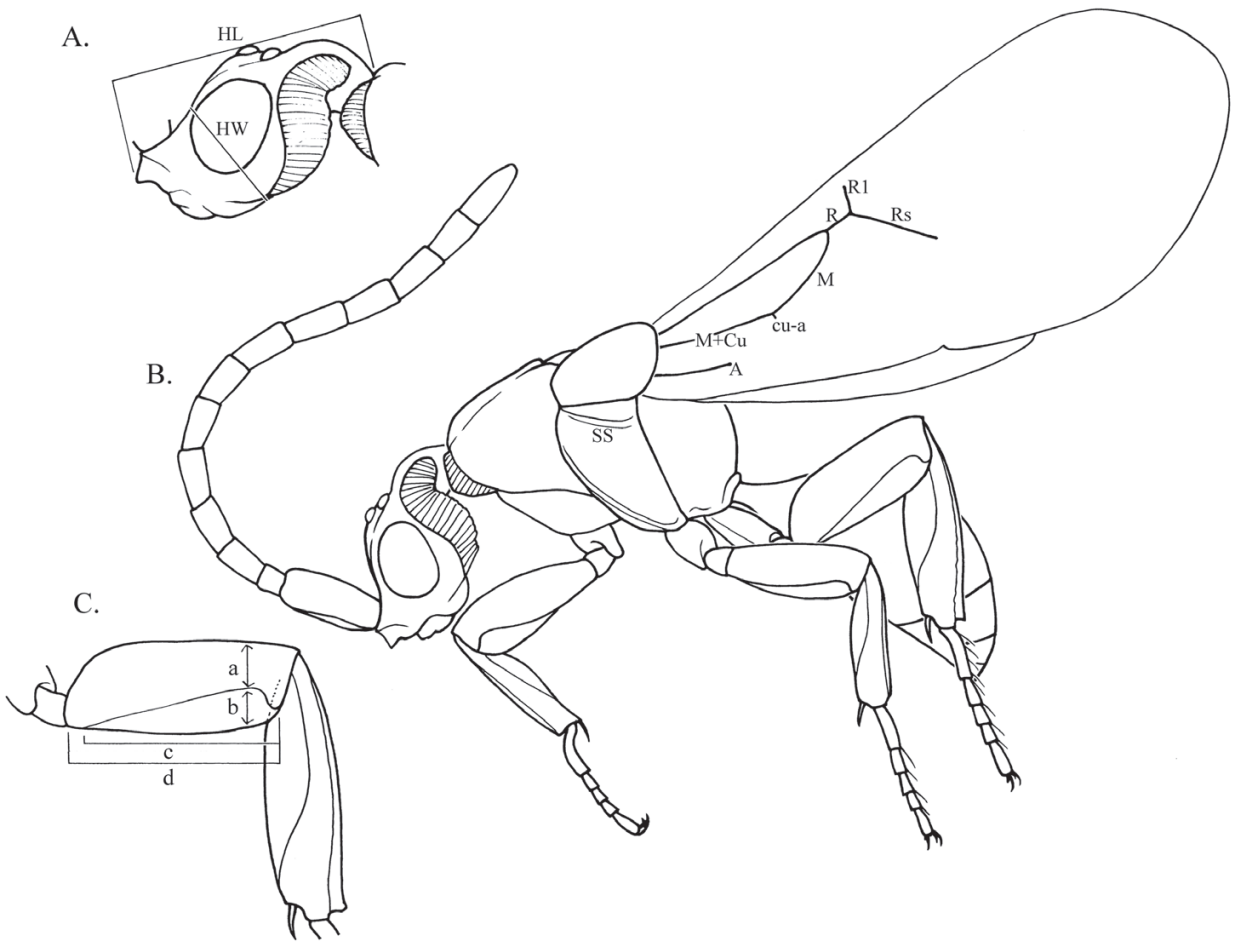
Diagram of lateral views of male *Loboscelidia pecki*. **A** Head, antenna removed. **B** Habitus of body. **C** Hindleg: (**a**) tubular part of femur width (**b**) femoral flange width (**c**) femoral flange length (**d**) femoral length. Abbreviations: **HL** = head length **HW** = head breadth **M+Cu** = media + cubital veins **M** = medial vein **cu-a** = cubital-anal cross vein **R** = radial vein **R1** = first radial branch **Rs** = radial sector **SS** = scrobal sulcus.

Loboscelidiines are characterized by a number of unusual features (Figs 1, 2). The antennae insert horizontally on a shelf-like extension in the middle of the face (the shelf-like extension is termed the frontal projection below); the vertex is prolonged posteriorly into a neck-like projection fringed with ribbon-like setae; the pronotum is not freely hinged to the scutum and has a short line of ribbon-like setae along the anterolateral corner; the tegula is very large, covering both wing bases, and is held in place by a ridge on the mesopleuron; the mesopleuron is smooth without sculpturing, except for a shallow, trough-like scrobal sulcus in some species, the propodeum lacks a dorsal surface and has an ear-like lateral projection over the spiracle, and the forewing lacks a stigma, costal and subcostal veins.

Distinctions between *Loboscelidia* and *Rhadinoscelidia* have been summarized in Kimsey and Bohart (1991). Briefly *Loboscelidia* can be distinguished from *Rhadinoscelidia* by the forewing venation extending into the basal one-third to one half of the wing (considerably less than one-fourth in *Rhadinoscelidia*), vertex convex or flat behind the ocelli, not sharply declivitous as in *Rhadinoscelidia*, and cervical expansion continuous with head, without discrete posterior expansion and with well-developed genal and cervical fringe. Cervical expansion basally constricted and shield-like posteriorly, with small discontinuous genal and cervical fringes in *Rhadinoscelidia*.

Members of the genus *Loboscelidia* are strongly sexually dimorphic, which has led to confusion over generic placement and sex associations. The genus *Scelidoloba* Maa & Yoshimoto, 1961 was erected for what turned out to be female *Loboscelidia* (Day 1978). Males have five external metasomal segments and a long slender flagellum. Females are heavier bodied than the males, with a shorter, broader flagellum and an externally four-segmented metasoma. It's not clear how many characteristics are shared between the two sexes as fewer than 15% of specimens in collections are female and more than one species may be present in a single locality. However, the sexes do seem to share some modifications of the wing venation (presence and shape, or absence of the medial vein), shape of the frontal projection, and presence or absence of the scrobal sulcus and notauli.

Little is known of the biology of the Loboscelidiinae. Specimens are rare in collections. However, this situation is probably more a reflection of collecting techniques used and sites visited than any indication of abundance. Malaise trapping in Thailand as part of the National Science Foundation funded TIGER project has yielded more than 100 *Loboscelidia* specimens, more than all other museum holdings. The small number of female *Loboscelidia* collected relative to males may be due to their differing habits. Males may be more frequently caught in traps because they tend to frequent low vegetation and the surface of leaf litter searching for females. Females may spend most of their time in cryptic situations, for example under bark or in the leaf litter, searching for hosts.

The morphology of the female ovipositor and mandibles closely resembles that of the Amiseginae, suggesting that loboscelidiines, like amisegines parasitize walking stick eggs. There is one report of an unidentified species of *Loboscelidia* reared from the eggs of the phasmatid *Acrophylla* sp. (Riek 1970). It is also possible, given the structural modifications of the group, including the leg and antennal flanges, the very large tegula and the tegular clip that *Loboscelidia* females at least may search for walking stick eggs in ant nests. Fouts (1922) suggested that the group is myrmecophilous based on the odd morphology. Walking stick eggs may be collected by ants because of the egg's strong resemblance to seeds.

**Figure 3.**
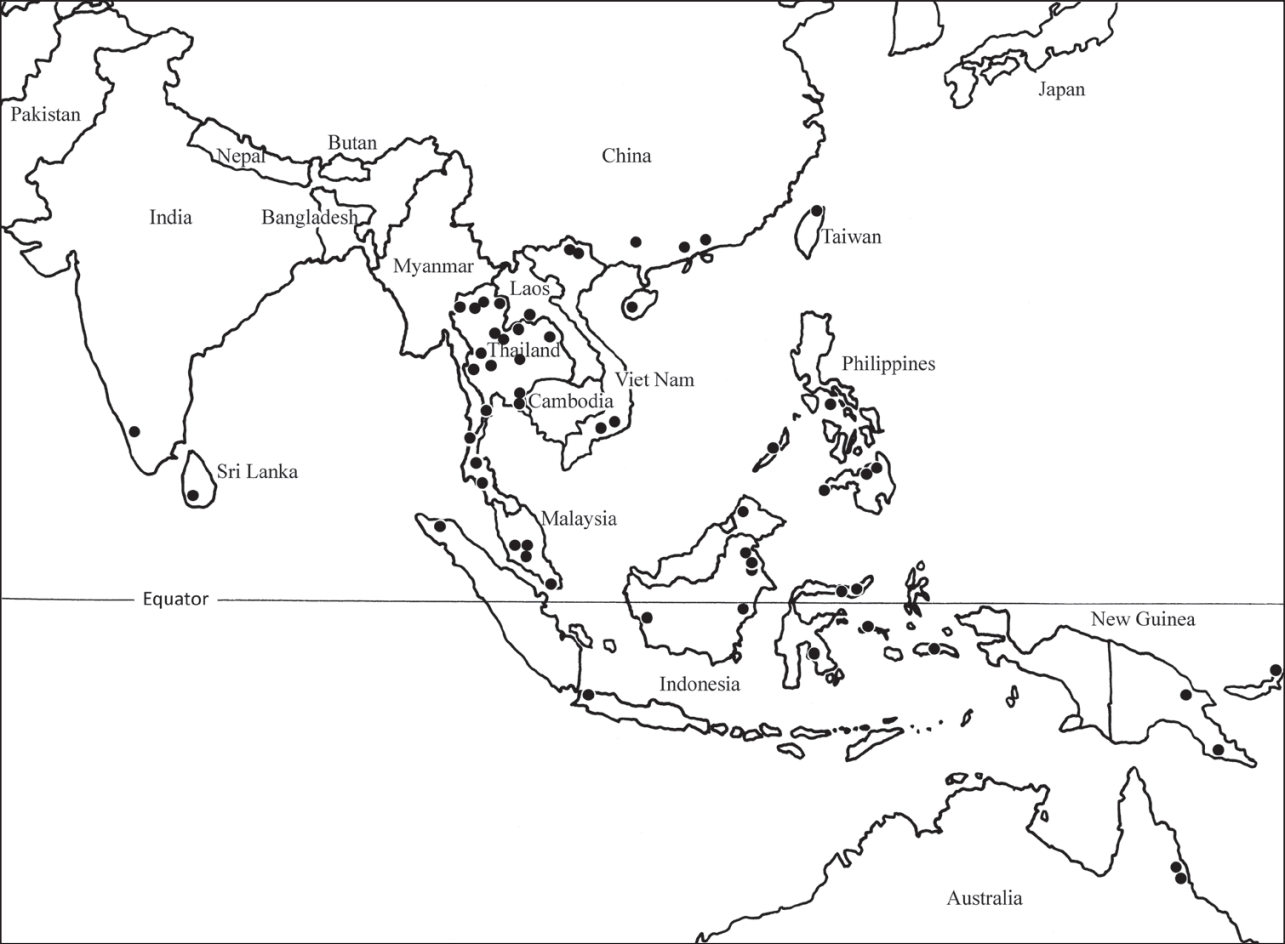
Distribution map of the genus *Loboscelidia* in south Asia and Australia.

## Materials and methods

Specimens were borrowed from the following museums, and type repositories are indicated by the acronyms: AEI – American Entomological Institute, Gainesville, Florida); ANIC – Australian National Insect Collection; BME – Bohart Museum of Entomology, University of California, Davis, USA; BMNH – The Natural History Museum, London, UK; BPBM – Bishop Museum, Honolulu, Hawaii, USA; CAS – California Academy of Sciences, San Francisco, USA, CNC – Canadian National Insect Collection, Ottawa, Ontario, Canada; CSIRO, Canberra, Australia, Australian National Insect Collection; MNHN – Museum National d'Histoire Naturelle, Paris; QSBG – Chiang Mai Royal Botanical Garden, Chiang Mai, Thailand; ROM – Royal Ontario Museum, Toronto, Canada; UCR – Entomological Research Museum, University of California, Riverside, USA, and USNM – U.S. National Museum, Washington, D.C., USA.

Additional type repositories include: CASB - Institute of Zoology, etc.; Institute of Zoology, Beijing, China; MZB – Museum Zoologicum Bogoriense Cibinong, Indonesia; NMNS – National Museum of Natural Science, Taichung, Taiwan; OUMNH – Oxford University Museum of Natural History, Oxford, UK; QDPI – CSIRO Long Pocket Laboratories, Indooroopily, Queensland, Australia; SCAC – Hymenoptera Collection, South China Agricultural University, Guangzhou, and ZFCL – Hymenoptera Collection, Zhejiang University, Hangzhou, China.

Morphological terminology follows that used by Kimsey and Bohart (1991) and is further described in Fig. 2. The hindwing lacks venation, so wing vein characters are only for the forewing. Wing veins are given in the text as abbreviations: Cu = cubital vein, cu-a = cubital-anal cross vein, M = medial vein, R = radial vein, Rs = radial sector, R1 = first radial branch. Scrobal sulcus refers to the transverse trough on the mesopleuron below the forewing ending in the scrobe adjacent to the metapleuron. The shape of the frontal projection is determined viewed in front view. It is considered triangular if the ventral angle of the projection ends in a point or the flat surface is less than one-tenth the length of the upper surface. The projection is considered rectangular if it is a true rectangle or rhomboid. Head length versus width is measured from the apex of the cervical extension to the furthermost point of the frontal projection and across the widest part of the head in lateral view. Antennal articles are measured at the point of greatest breadth and compared with the total length of the article. Wing veins are compared relative to the length of R1. Pronotal dimensions are measured from the medial length of the pronotum in dorsal view to the distance between the apices of the posterolateral angles. The length of a leg flange is measured from the basal joint to the apex of the segment along the ventral margin. The relative width of leg flanges are measured across the broadest part of the flange relative to the tubular part of the segment at the same point.

### Key to males of the species of *Loboscelidia*

**Table:** 

1	M vein incomplete medially or absent (as in Figs 24, 26, 28, 35)	2
–	M vein complete	6
2	M vein incomplete medially, Rs twice as long as R (Fig. 28); India	*Loboscelidia incompleta* sp. n.
–	M vein absent, Rs less than twice as long (as in Fig. 26) or 2.5× as long as R	3
3	Propodeum broadly angulate dorsomedially in posterior view; Borneo	*Loboscelidia bakeri* Fouts
–	Propodeum flat to gently convex dorsally in posterior view	4
4	Fore, mid and hindtibiae without measurable flanges (Fig. 46); Laos, Viet Nam; Thailand	*Loboscelidia reducta* Maa & Yoshimoto
–	Fore, mid and hindtibiae with flanges 0.9× as long and 0.3-1.0× as wide as tubular part of respective tibia	5
5	Rs less than 1.5× as long as R, A less than 0.5× as long as Cu+M (Fig. 27); Viet Nam	*Loboscelidia fulgens* sp. n.
–	Rs more than twice as long as R, A 0.9–1.1× as long as Cu+M; China	*Loboscelidia guangxiensis* Xu
6	Gena and often legs with scattered scale-like setae (as in Fig. 16)	7
–	Gena and legs without scale-like setae	8
7	M straight medially (Fig. 36); scape less than 3× as long as broad; Borneo	*Loboscelidia sisik* Kimsey sp. n.
–	M curved submedially; scape more than 3× as long as broad; Viet Nam	*Loboscelidia asiana* Kimsey
8	Vertex extension flattened in lateral view, not depressed behind ocelli (as in Fig. 11); foretibia without transparent flange, except in *Loboscelidia nitidula* (as in Fig. 45)	9
–	Vertex extension convex in lateral view, depressed behind ocelli (as in Fig. 4); foretibial flange usually present	12
9	Tibial flanges well-developed (as in Fig. 45); scrobal sulcus present	10
–	Tibial flanges represented by posterior ridge or absent (as in Fig. 42); scrobal sulcus absent	11
10	Rs 3.2–4.0× as long as R; R1 and cu-a shorter than R (Fig. 33); Thailand	*Loboscelidia nitidula* sp. n.
–	Rs 2.5–3.0× as long as R or shorter; R1 and cu-a as long as R; Taiwan	*Loboscelidia latigena* Lin
11	Propodeum without transverse subapical carina; cu-a less than 0.3× as long as R; legs smooth, not striate; Borneo, Sumatra	*Loboscelidia brunnea* Fouts
–	Propodeum with transverse subapical carina; cu-a more than 0.5× as long as R; legs extensively longitudinally striate (Fig. 42); Borneo, Malaysia, Singapore, Sumatra	*Loboscelidia maculipennis* Fouts
12	M straight medially (as in Fig. 27)	13
–	M curved submedially	18
13	Scutum without notauli (as in Fig. 22)	14
–	Scutum with notauli (as in Figs 21, 23)	15
14	Hindfemoral flange 2.5× as wide as tubular part of femur; hindtibial flange twice as wide as tubular part of tibia; Australia	*Loboscelidia maculata* Kimsey
–	Hindfemoral flange twice as wide as tubular part of femur; hindtibal flange as wide as tubular part of tibia; Australia	*Loboscelidia ora* Kimsey
15	Scrobal sulcus present at least as a series of pits or foveae (as in Fig. 2); scape 3.0× as long as broad or shorter; cu-a 0.3× as long or longer than R (as in Fig. 27)	16
–	Scrobal sulcus absent; scape 3.5× as long or longer as broad; cu-a absent	17
16	Face frontal projection rhomboid or rectangular in front view; Rs 3.0× as long as R (Fig. 27); midtibial flange more than half as long and wide as tubular part of tibia (Fig. 39); Thailand, Sumatra	*Loboscelidia fulva* sp. n.
–	Face frontal projection triangular in front view; Rs 2.5× as long as broad or shorter (Fig. 31); midtibial flange absent or less than half as long and wide as tubular part of tibia (Fig. 43); Borneo	*Loboscelidia meifungae* sp. n.
17	Rs more than twice as long as R, more than 0.8× as long as M+Cu; Java	*Loboscelidia halimunensis* Kojima
–	Rs less than twice as long as R, A 0.5–0.7× as long as M+Cu; Philippines	*Loboscelidia defecta* Kieffer
18	Scutum without notauli or notauli about half as long as scutum (as in Figs 22, 23)	19
–	Scutum with notauli 0.7–1.0× scutal length	21
19	Scutum without notauli; face with frontal projection rhomboid in front view (as in Fig. 20); flagellomeres I-II each less than twice as long as broad; Australia	*Loboscelidia australis* Kimsey
–	Scutum with notauli about half as long as scutum; face with frontal projection linear to broadly triangular or V-shaped in front view (as in Fig. 19); flagellomeres I-II each twice or more as long as broad	20
20	Foretibia without transparent flange; hindfemoral flange half as wide as femur; Rs more than 3× as long as R; New Britain	*Loboscelidia cervix* Maa & Yoshimoto
–	Foretibia with transparent flange; hindfemoral flange as wide as femur; Rs less than 3× as long as R; New Britain	*Loboscelidia parva* Maa & Yoshimoto
21	Frontal projection nearly linear in front view (as in Fig. 18); cu-a as long as R	22
–	Frontal projection rectangular, rhomboid (as in Fig. 20) (extremely elongate in *Loboscelidia nasiformis*) or triangular (as in Fig. 19); cu-a shorter than R or absent	23
22	Foretibial flange half as wide as tubular part of tibia; midtibial flange half as long and half as wide as tubular part of tibia; New Guinea	*Loboscelidia novoguineana* Kimsey
–	Foretibial flange as wide as tubular part of tibia; midtibial flange 1.5× as long and as wide as tibia tubular part of; Australia	*Loboscelidia nigricephala* Kimsey
23	Face with frontal projection elongate and nasiform; head nearly 3× as long as broad (Fig. 13); Thailand	*Loboscelidia nasiformis* sp. n.
–	Face with frontal projection rectangular to triangular; head twice or less as long as broad	24
24	cu-a less than 0.2× as long as R or absent	25
–	cu-a 0.2–0.4× as long as R	26
25	R1 as long as R, Rs 3× as long as R (Fig. 34); Viet Nam	*Loboscelidia pecki* sp. n.
–	R1 absent or less than 0.4× as long as R, Rs less than 2.2× as long as R (Fig. 25); Thailand, Borneo, Singapore, Malaya	*Loboscelidia cinnamonea* sp. n.
26	Midfemoral flange 0.3× as long as femur; R1 less than 0.3× as long as R and A vein as long as Cu+M; China	*Loboscelidia sinensis* Kimsey
–	Midfemoral flange 0.4-1.0× as long as femur; R1 0.4–1.0× as long as R and A vein shorter than Cu+M, except in *Loboscelidia indica*	27
27	R1 reaching R at nearly right angle; pronotal length 0.4–0.6× width across posterolateral angles or shorter; China	*Loboscelidia levigata* Yao, Liu & Xu
–	R1 reaching R at obtuse angle; pronotal length greater than 0.6× width across posterolateral angles	28
28	Scrobal sulcus absent	29
–	Scrobal sulcus present (as in Fig. 2)	31
29	Propodeum with transverse subapical carina; metanotum less than 0.3× as long as scutellum; Borneo, Sula Is	*Loboscelidia nixoni* Day
–	Propodeum without transverse subapical carina; metanotum more than 0.3× as long as scutellum	30
30	Scape more than 3.0× as long as broad; hindtibial flange wider than tubular part of tibia; Philippines	*Loboscelidia philippinensis* Fouts
–	Scape less than 3.0× as long as broad; hindtibial flange narrower than tubular part of tibia; Borneo, Sula Is	*Loboscelidia rufescens* Westwood
31	Frontal projection triangular (as in Fig. 19)	32
–	Frontal projection rhomboid or rectangular (as in Fig. 20)	37
32	Rs more than 3.0× as long as R; flagellomere I less than twice as long as broad; Laos, Sumatra	*Loboscelidia laotiana* Kimsey
–	Rs 2.5–3.0× or less as long as R; flagellomere I twice or more as long as broad	33
33	Flagellomere XI more than 4.0× as long as broad	34
–	Flagellomere XI 4.0× or less as long as broad	35
34	Scape less than 3× as long as broad; forefemoral flange half as wide as tubular part of femur; hindtibial flange as wide as tubular part of tibia or narrower; Philippines	*Loboscelidia nigra* Fouts
–	Scape more than 3× as long as broad; forefemoral flange as wide as tubular part of femur; hindtibial flange twice as wide as tubular part of tibia; Sri Lanka	*Loboscelidia castanea* Krombein
35	Hindtibial flange less than 1.5× as wide as tubular part of tibia; flagellomere XI less than 3× as long as broad; Philippines	*Loboscelidia scutellata* Fouts
–	Hindtibial flange more than 1.5× as long as wide as tubular part of tibia; flagellomere XI more than 3× as long as broad	36
36	Hindtibial flange 2.0–2.5× as wide as tubular part of tibia (as in Fig. 41); Singapore	*Loboscelidia collaris* Fouts
–	Hindtibial flange less than twice as wide as tubular part of tibia (as in Fig. 40); Borneo, Sulawesi	*Loboscelidia sarawakensis* Kimsey
37	Scape 3.9–4.1× as long as broad, flagellomere XI 3.9–4.1× as long as broad; Philippines	*Loboscelidia rufa* Fouts
–	Scape less than 3.8× as long as broad; flagellomere XI less than 3.8× as long as broad	38
38	Foretibial flange narrower than tubular part of tibia (as in Fig. 40)	39
–	Foretibial flange as wide or wider than tubular part of tibia (as in Fig. 41)	40
39	Rs twice as long as R; scape 3× as long as broad; flagellomere I twice as long as broad; Sri Lanka	*Loboscelidia atra* Krombein
–	Rs 3× as long as R; scape less than 3× as long as broad; flagellomere I less than twice as long as broad; Viet Nam, Thailand	*Loboscelidia laminata* sp. n.
40	Fore and midtibial flanges as wide as or narrower than tubular part of respective tibiae; Thailand, Laos, Viet Nam, Malaya, Borneo	*Loboscelidia kafae* sp. n.
–	Fore and midtibial flanges more than 1.2× as wide as tubular part of respective tibiae	41
41	A longer than Cu-M; Rs less than 3.0× as long as R; pronotum rounded laterally; India	*Loboscelidia indica* Kimsey
–	A shorter than Cu-M; Rs 3.4× as long as R; pronotum with carinate lateral edge; Borneo, Thailand	*Loboscelidia pasohana* Kimsey

## Species treatments

### Loboscelidia antennata

Fouts

http://species-id.net/wiki/Loboscelidia_antennata

Loboscelidia antennata Fouts 1922: 622. Holotype female; Singapore (USNM).

#### Material studied.

Singapore (USNM); Indonesia: West Kalimantan, Gunung Palung National Park (1 female, ROM); 2 female specimens were examined including the holotype.

#### Diagnosis.

The male of this species is unknown, but *Loboscelidia antennata* may very well prove to be the female of *Loboscelidia brunnea* Fouts, based on the triangular frontal projection, flattened cervical expansion, curved medial vein and lack of a scrobal sulcus.

### Loboscelidia asiana

Kimsey

http://species-id.net/wiki/Loboscelidia_asiana

Loboscelidia asiana Kimsey 1988: 68. Holotype male; Viet Nam: Dalat (BPBM).

#### Material studied.

Only the holotype was seen.

#### Diagnosis.

The most distinctive feature of *Loboscelidia asiana* is the presence of spatulate or leaf-like setae on the gena, a character shared only with *Loboscelidia sisik* (as in fig. 16). However, *Loboscelidia asiana* can be distinguished from *Loboscelidia sisik* by the submedially curved medial vein (nearly flat in *Loboscelidia sisik*), scape striate and more than 3.5× as long as broad (smooth and less than 3× as long as broad in *Loboscelidia sisik*) and no scrobal sulcus (present in *Loboscelidia sisik*).

### Loboscelidia atra

Krombein

http://species-id.net/wiki/Loboscelidia_atra

Loboscelidia atra Krombein 1983: 52. Holotype male; Sri Lanka: Sabaragamuwa Prov., Ratnapura Dist., Sinharaja Jungle (USNM).

#### Material studied.

Only the holotype was seen.

#### Diagnosis.

This is one of several species with a well-developed, complete scrobal sulcus. A combination of features will separate *Loboscelidia atra* from these other species, including the rectangular frontal projection (in lateral view), scape more than 3× as long as broad, cu-a vein less than half as long as R, Rs twice as long as R, and metanotum half as long as the scutellum.

### Loboscelidia australis

Kimsey

http://species-id.net/wiki/Loboscelidia_australis

Figure 22

Loboscelidia australis Kimsey 1988: 69. Holotype male; Australia: NSW (AEI).

#### Material studied.

Australia: New South Wales, Queensland; two specimens were seen including the holotype.

#### Diagnosis.

This is one of three species (including *Loboscelidia maculata* and *Loboscelidia ora*), all Australian, that lack notauli (as in Fig. 22). *Loboscelidia australis* can be distinguished from these by the submedially curved medial vein, rectangular frontal projection, pronotum with sharp lateral fold or ridge, flagellomere XI less than 3× as long as broad, and fore and midtibial flanges less than 0.5× as long as their respective tibial lengths.

### Loboscelidia bakeri

Fouts

http://species-id.net/wiki/Loboscelidia_bakeri

Figure 24

Loboscelidia bakeri Fouts 1922: 620. Syntype males (not females) (3); Borneo: Sandakan (USNM).

#### Material studied.

Malaysian Borneo, Sabah, Sandakan (2 males, USNM), Kinabalu National Park Poring Hot Springs (2 males including two syntypes, CNC, USNM).

**Diagnosis.** *Loboscelidia bakeri* can be immediately distinguished from all other *Loboscelidia* species by the distinctively dorsomedially up-domed propodeum. It is also one of four species, including *Loboscelidia fulgens*, *Loboscelidia reducta* and *Loboscelidia ganxiensis* that lack a medial vein (as in Fig. 24).

### Loboscelidia brunnea

Fouts

http://species-id.net/wiki/Loboscelidia_brunnea

Loboscelidia brunnea Fouts 1922: 626. Holotype male (not female); Borneo: Sandakan (USNM).

#### Material studied.

Malaysian Borneo, Sabah; only the holotype was seen.

#### Diagnosis.

Four *Loboscelidia* species, *Loboscelidia brunnea*, *Loboscelidia maai*, *Loboscelidia nitidula* and *Loboscelidia maculipennis*, have a strongly flattened cervical expansion. *Loboscelidia brunnea* can be distinguished from theseby the extreme reduction of cu-a, Rs vein less than 3.5× as long as R, the legs coarsely striate, and hindtibial posterior margin essentially ecarinate.

### Loboscelidia castanea

Krombein

http://species-id.net/wiki/Loboscelidia_castanea

Loboscelidia castanea Krombein 1983: 54. Holotype male; Sri Lanka: Sabaragamuwa Prov., Ratnapura Dist., Sinharaja Jungle (USNM).

#### Material studied.

Sri Lanka, Sabaragamuwa Prov.; only the holotype was seen.

#### Diagnosis. 

This is one of the species with a complete scrobal sulcus and triangular frontal projection. It shares a long scape (more than 3× as long as broad) with one of these, *Loboscelidia laotiana*. *Loboscelidia castanea* can be distinguished from these species and *Loboscelidia laotiana* by a combination of characters, including cu-a less than 0.5× as long as R, A 0.6× as long as Cu+M, flagellomere I shorter than II, flagellomere XI more than 4× as long as broad, and the fore, mid and hindfemoral flanges as broad as the tubular part of the respective femora.

### Loboscelidia cervix

Maa & Yoshimoto

http://species-id.net/wiki/Loboscelidia_cervix

Figure 23

Loboscelidia cervix Maa and Yoshimoto 1961: 546. Holotype male; New Britain: Vudal, near Keravat (BPBM).

#### Material studied.

New Britain: near Keravat only the holotype was seen.

#### Diagnosis.

This is one of two species, including *Loboscelidia parva*, known from New Britain. Both have the notauli not reaching the posterior margin of the scutum (Fig. 23) and the frontal projection sublinear in front view. *Loboscelidia cervix* can be distinguished from *Loboscelidia parva* by the shorter scape (2.6–2.8× as long as broad in *Loboscelidia cervix*, 3.0–3.1× in *Loboscelidia parva*), Rs more than 3× as long as R (less than 3× in *Loboscelidia parva*), cu-a longer than R (shorter in *Loboscelidia parva*) and partial scrobal sulcus (absent in *Loboscelidia parva*). The Australian species *Loboscelidia ora* is the only other *Loboscelidia* with long cu-a longer than R.

### Loboscelidia cinnamonea sp. n.

urn:lsid:zoobank.org:act:E5A2B8FA-4264-468B-B3A5-B52456903906

http://species-id.net/wiki/Loboscelidia_cinnamonea

Figures 4 25 37

#### Type material.

Holotype male: Thailand: **Chiang Mai Pr.**, Doi Chiangdao NP, Pha Tang substation, 526 m, 19°24.978"N, 98°54.886"E, Malaise trap, 3-9/v/2008, Jugsu & Watwanich, T5802 (QSBG).

Paratypes (25 males): 3 males, same data as type; 1 male: Doi Chiangdao NP, 491 m, 19°24.278'N, 98°55.311'E, Malaise trap, 15–21/v/2008, Jugsu & Watwanich, T5815; 1 male: Doi Chiangdao NP, Pha Tang substation, 491 m, 19°24.278'N, 98°55.311'E, Malaise trap, 9–15/v/2008, Jugsu & Watwanich, T5812; 2 males: Doi Chiangdao NP, Huai Na Lao, 500m, 19°24.731'N, 98°55.315'E, YPT 5-6/v/2008, Jugsu & Watwanich, T5806; 1 male: Doi Chiangdao NP, Huai Na Lao, 500m, 19°24.731'N, 98°55.315'E, YPT 9-10/v/2008, Jugsu & Watwanich, T5811; 1 male: Doi Chiangdao NP, Huai Na Lao, 500m, 19°24.731'N, 98°55.315'E, YPT 4-5/v/2008, Jugsu & Watwanich, T5805; 2 males: **Lampang Pr.**, Chae Son NP, Doi Laan, 18°51.815'N, 99°22.122'E, 1413 m, Malaise trap, 9-15/v/2008, Kwannui & Sukpeng, T5292; 1 male: Chae Son NP, 18°49.894'N, 99°28.354'E, 467 m, Malaise trap, 23–30/v/2008, Kwannui & Sukpeng, T5305; 1 male: Chae Son NP, 18°50.012'N, 98°28.656'E, 419 m, pan trap, 7-8/v/2008, Kwannui & Sukpeng, T5304; 3 males: Chae Son NP, 18°49.894'N, 99°28.354'E, 467 m, Malaise trap, 1-7/v/2008, Kwannui & Sukpeng, T5309; 1 male: **Chanthaburi Pr.**, Khao Khitchakut NP, Khao Prabaht peak, 12°50.45'N, 102°09.81'E, 875 m, Malaise trap, 20–27/ii/2009, Suthida & Charoenchai, T4045; 1 male: Khao Khitchakut NP, Khao Prabaht peak, 12°50.45'N, 102°09.81'E, 875 m, Malaise trap, 6-13/ii/2009, Suthida & Charoenchai, T4039; 1 male: **Trang Prov.**, Khaeochong Mt, 75 m, 7°33.038'N, 99°47.369'E, Malaise, 28/iv-2/c/2005; 2 males: near Nam Tock Ton Prov., Khoa Chong Mt.,140 m, 7°32.015'N, 99°47.036'E, iv/2005 and ii/2005; 1 male: **Phetchabun Pr.**, Nam Nao NP, 16°43.695'N, 101°33.797'E, 921 m, Malaise trap, 5-12/v/2007, L. Janteab, T2657; 1 male: **Kanchanaburi Pr.**, Khuean Srinagarindra NP, 14°38.136'N, 98°59.837'E, 210 m, pan trap, 21-22/viii/2008, Chatchawan, T3438; 1 male: **Sakon Pr.**, Nakhon Phu Phan NP, 17°03.543'N, 103°58.452'E, 8-14/vii/2006, MT, W. Kongnara, T197; 1 male: **Suphan Buri Pr.**, Khao Yai NP, Kong Geo waterfalls, 900 m, 30/vi/1990, J. Heraty, H90/108. Paratypes are deposited in QSBG and BME.

Additional non-type specimens (27) were seen from: Borneo: north, Tawa, Quoin Hill (1 male, BPB); Sabah: Kinabalu Nat. Park, Poring Hot Springs (4 males, CNC); Sarawak: sw Gunung Buda, 64 km s Limbang (BME); W. Kalimantan: Gunung Palung Nat. Pk. (3 males, ROM, BME); E. Kalimantan: Kac. Pujungan, Kayan-Mantarang Nat. Res. (1 male, ROM); West Java: Gede-Pangrango Nat. Park, Situ Gunung (2 males, ROM, BME); Sumatra: Aceh, Gunung Leuser Nat. Pk. (1 male, ROM); Malaysia: Selangor (1 male, UCR);Pahang: Kuala Tahan, Taman Negara Nat. Park (1 male, UCR); Malaya: 10 mi e Gombak (1 male, UCR); Thailand: Mae Hong Son, Namtok Mae Surin Nat. Pk (1 male, QSBG); Nakon Si Thammarat:Namtok Yong Nat. Pk. (1 male, QSBG); Phang Na: Khuraburi Dist. south end of Koh Res. (1 male, UCR); Trang: Forest Res. Sta. Khao Chong (1 male, UCR); Singapore (7 males, BPBM, UCR).

#### Diagnosis.

*Loboscelidia cinnamonea* is most similar to *Loboscelidia nasiformis*, as both share an arched medial vein, rectangular frontal projection, complete notauli, without a scrobal sulcus and the cu-a vein reduced to a tiny stub or absent. It can be distinguished from *Loboscelidia nasiformis* by the more typical frontal projection, fore and midtibiae without discrete, measureable flanges, R1 obsolescent and Rs 3× or more as long as R.

#### Male description.

**Figures 4–13.**
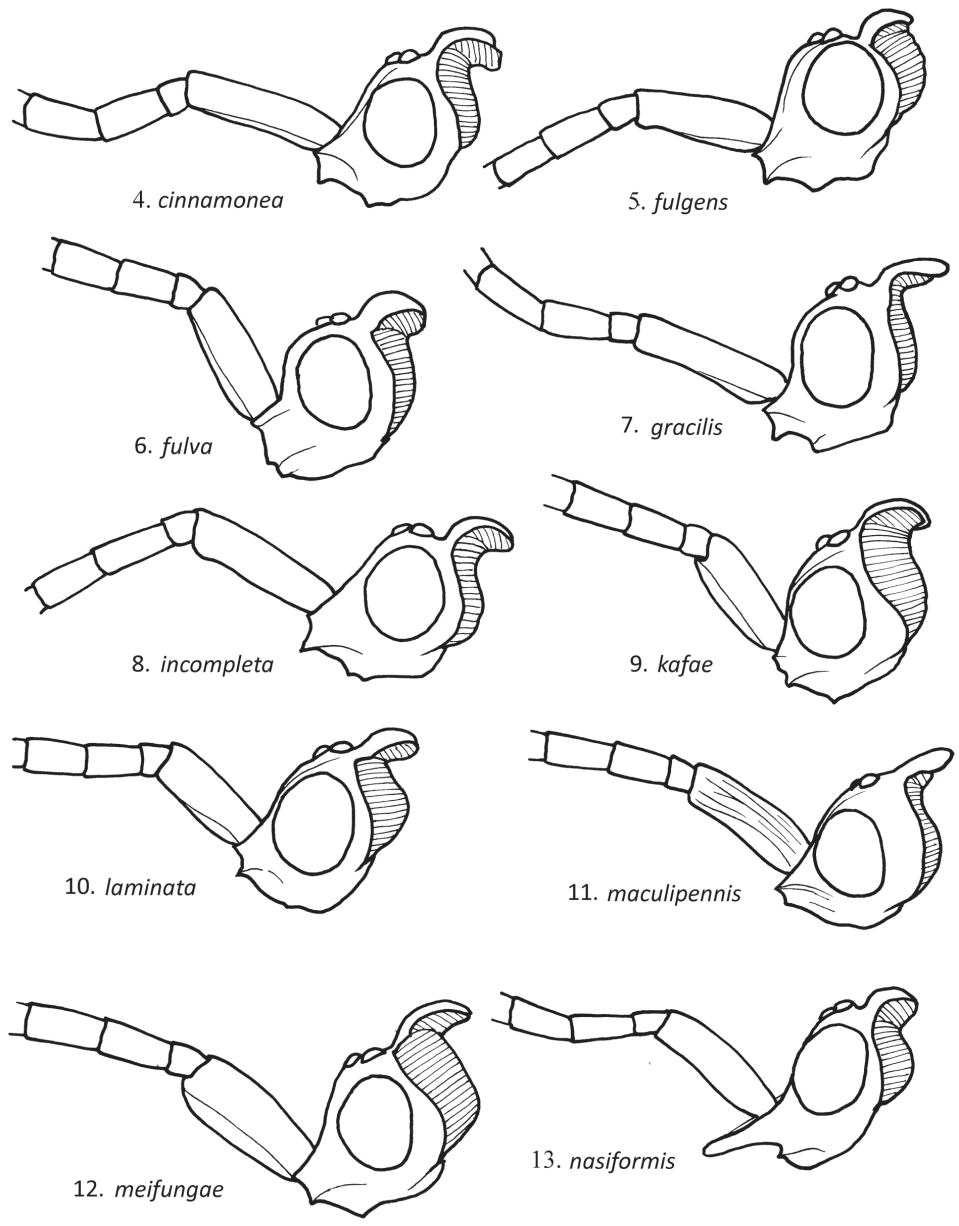
Lateral view of male *Loboscelidia* head, with basal antennal segments.

Body length 2.0–3.0 mm; forewing length 2.5–3.5 mm. Head (Fig. 4): length twice breadth in side view; eye asetose; frontal projection rectangular in front view; frons smooth, not microstriate; frons with low ridge extending from vertex along inner eye margin; vertex without transverse fovea, cervical expansion strongly curved in profile; gena without scale-like setae; scape smooth, length 3.9 breadth; flagellomere I length 2× breadth; flagellomere II length 2.3× breadth; flagellomere XI length 5× breadth. Mesosoma: pronotal length 0.9× breadth, without lateral carina, pronotum narrower than head width; scutum with notauli reaching posterior margin; scutellum with sublateral carina, without fine dense striae laterally; metanotum without medial ridge, impunctate laterally, 0.4× as long as scutellum; mesopleuron without scrobal sulcus; propodeum without transverse dorsal carina; legs (Fig. 37) smooth, polished; forefemoral flange 0.4 x femur length, flange maximum width equal to width of tubular part of femur; foretibial flange absent; midfemoral flange 0.6× femur length, flange maximum width 0.6× width of tubular part of femur; midtibial flange absent; hindfemoral flange 0.9× femur length, flange maximum width 0.7× width of tubular part of femur; hindtibial flange as long as tibia, flange maximum width 0.8× width of tubular part of tibia; hindtibia with two longitudinal carinae on posterior margin; hindcoxa without longitudinal carina on inner medial surface; forewing (Fig. 25) R1 length 0-0.2× R length; cu-a length 0.1× R length; Rs length twice R length; Cu+M length 0.4-0.6× A length; medial vein curved submedially. Color: body reddish brown to dark orange; wing membrane brown-tinted, with untinted areas adjacent to vein remnants; veins brown.

#### Female.

Unknown.

**Figures 14–26:**
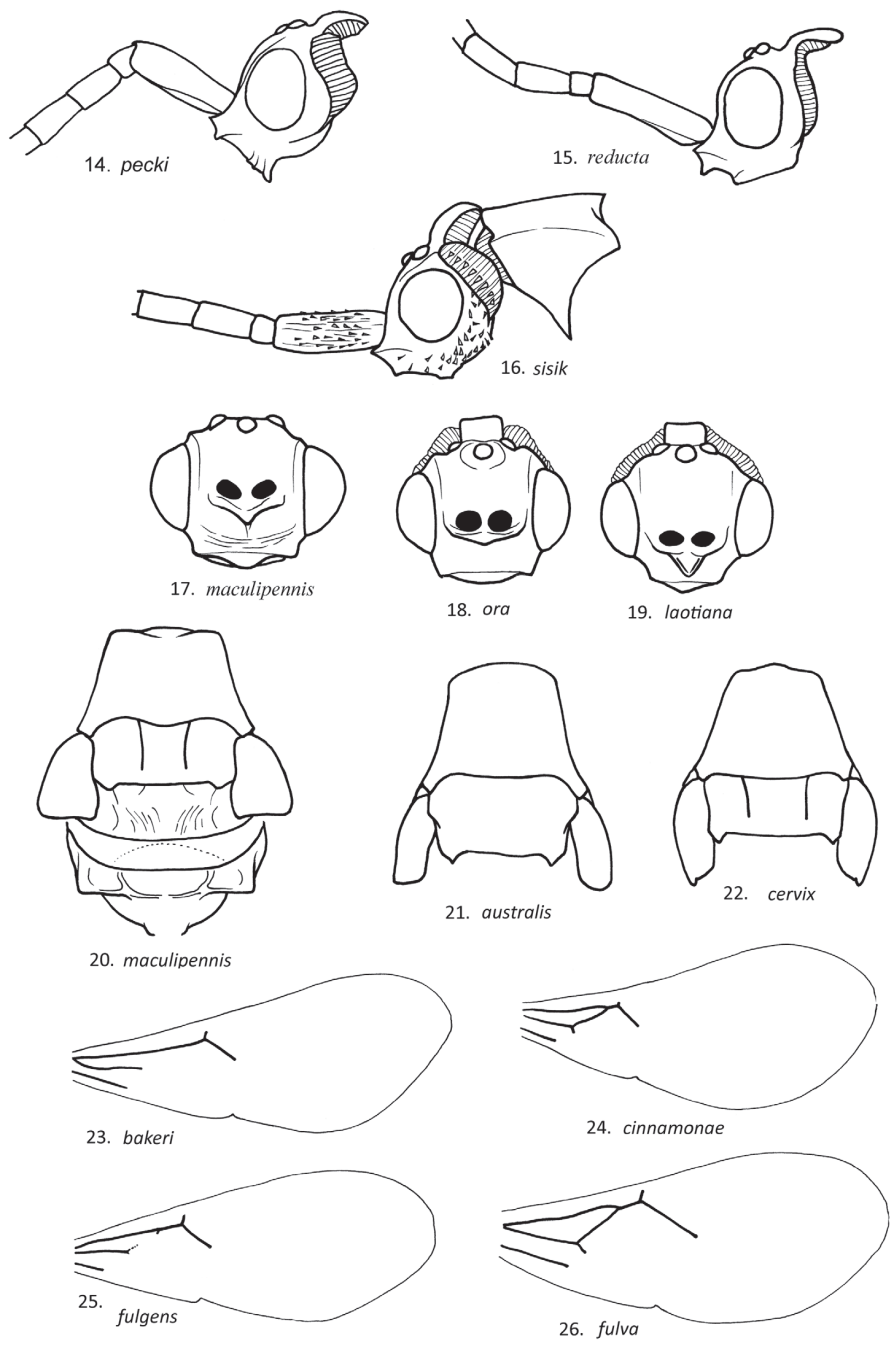
. Lateral view of male *Loboscelidia* head, with basal antennal segments. **17–19**. Front view of face with antennae removed **20** Dorsal view of thorax, with wings removed **21, 22** Dorsal view of pronotum scutum and tegulae **23–26** Forewings.

#### Etymology.

The species name is Latin for brown as in the spice, cinnamon.

### Loboscelidia collaris

Fouts

http://species-id.net/wiki/Loboscelidia_collaris

Loboscelidia collaris Fouts 1922: 627. Holotype male (not female); Singapore (USNM).

#### Material studied.

Indonesia: W. Kalimantan: Gunung Palung Nat. Pk (14 males, ROM; E. Kalimantan: Kac. Pujungan, Kayan-Matanrang Nat. Res. (3 males, ROM, BME); 38 km n alikpapan, Sambojal2 (1 male, ROM); Sumatra: Aceh, Gunung Leuser Nat. Park, Ketambe Res. Sta. (7 males, ROM, BME); Malaysia: Sabah, Mt. Kinabalu N.P., Poring Hot Spgs (2 males, CNC); Sarawak: Gunung Mulu National Park (4 males, BME, ROM); Selangor: 16 mi e Gombak, Univ. Malaya Forest (1 male, UCR); Singapore: (1 male, USNM), Timah Nat. Res. (1 male, CNC);; Thailand: Chaiyaphum,Tat Tone NP (1 male, QSBG); Trang: Near Nam Tock Tjon Prov., Khoa Chong Mt. (3 males, CNC); Phattalung Nam Tok Phrai Wan (1 male, UCR); 40 specimens were examined including the holotype.

#### Diagnosis.

This is another species with a complete scrobal sulcus and triangular frontal projection. Male *Loboscelidia collaris* can be distinguished from species with these traits by the combination of the pronotum with a sharp crease or ridge dorsolaterally, scape less than 3× as long as broad, flagellomeres I and II more than twice as long as broad, flagellomere XI 3.5–4.0× as long as broad, and the fore, mid and hindfemoral flanges as long as the femora.

### Loboscelidia defecta

Kieffer

http://species-id.net/wiki/Loboscelidia_defecta

Loboscelidia defecta Kieffer 1916b: 18. Syntype male, female; Philippines: Palawan (Insel Palavan), Puerto Princesa (MNHN, lost?).

#### Material studied.

Viet Nam: Karyu Danar (1 male, BPBM), Thailand: Mae Hong Son Pr., Namtok Mae Surin NP (1 male, BME); Nakhon Si Pr., Thammarat Namtok Yong (1 male, QSBG); Surat Thani Pr., Khao Sok Np, Klong Morg unit (1 male, BME); Chiang Mai Pr., Doi Chiangdao NP (1 male, QSBG); Malaysia: Sarawak, Gunung Lulu National Park (1 male ROM); 6 specimens were seen that appear to fit the original description.

#### Diagnosis.

The types of *Loboscelidia defecta* are apparently lost. However, based on Kieffer's (1916a) illustration it is one of the species that lacks a cu-a vein. In the same paper Kieffer attributed *Loboscelidia defecta* and *Loboscelidia inermis* to a 1915 paper he gives in the 1916a paper as “Philippine J. Sci. v. 10 p?”, but there was evidently no paper published by Kieffer in 1915 in volume 10 of this journal. Instead, *Loboscelidia defecta* Kieffer and *Loboscelidia inermis* Kieffer were published as new species one month after the 1916a paper (1916b). The 1915 date may have been a mistake on his part caused by delays in publication of the description paper in the Philippine Journal of Science.

### Loboscelidia fulgens sp. n.

urn:lsid:zoobank.org:act:229B3296-7FD3-49E4-8626-590CD8CDC23E

http://species-id.net/wiki/Loboscelidia_fulgens

Figs 5 26 38

#### Type material.

Holotype male: Viet Nam: Tuyen Quang Prov., 360 m, Na Hang Reserve, 16–20 May 1997, FIT, S. B. Peck, 97-10 (CNC). Paratypes: 3 males same data as holotype; 1 male: 20-24 May 1997, 97-13; 1 male: 300 m, 97-17; 1 male: Ha Tinh, Huong Son, 450 m, 18°22'N, 105°13'E, 22 April-1 May 1998, L. Herman, LT (BME, CNC).

#### Diagnosis.

This is one of four species, including *Loboscelidia bakeri*, *Loboscelidia guangxiensis* and *Loboscelidia reducta* that completely lack a medial vein. *Loboscelidia fulgens* can be separated from *Loboscelidia guangxiensis* in males by the shorter Rs vein, 1.5× as long as R, versus twice as long in *Loboscelidia guangxiensis*, and having well-developed tibial flanges, which are lacking in *Loboscelidia reducta*. *Loboscelidia fulgens* can be immediately distinguished from *Loboscelidia bakeri* by lacking the uniquely up-domed propodeum characteristic of *Loboscelidia bakeri*.

#### Male description.

Body length 1.5–2.0 mm; forewing length 2.0–2.5 mm. Head (Fig. 5): length 1.8× height in side view; eye asetose; frontal projection rectangular in front view; frons smooth, not microstriate; frons with low ridge extending from vertex along inner eye margin; vertex with transverse fovea, posterior expansion strongly curved in profile; gena without scale-like setae; scape striate, length 2.9× breadth; flagellomere I length 2× breadth; flagellomere II length 1.8× breadth; flagellomere XI length 3× breadth. Mesosoma: pronotal length 0.8× breadth, without lateral carina, narrower than head in dorsal view; scutum with notauli reaching posterior margin; scutellum with fine dense striae laterally; metanotum with medial ridge, impunctate laterally, 0.4× as long as scutellum; mesopleuron without scrobal sulcus; propodeum without transverse dorsal carina; legs (Fig. 38) smooth, polished; forefemoral flange 0.5× femur length, flange maximum width 0.6× width of tubular part of femur; foretibial flange 0.5× tibial length, flange maximum width 0.4 x width of tubular part of tibia; midfemoral flange absent; midtibial flange 0.6× femur length, flange maximum width 0.5× width of tubular part of tibia; hindfemoral flange 0.8× femur length, flange maximum width 0.6× width of tubular part of femur; hindtibial flange 0.8× femur length, flange maximum width 0.7× width of tubular part of tibia; hindtibia with two longitudinal carinae on posterior margin; hindcoxa without longitudinal carina on inner medial surface; forewing (Fig. 26) R1 length 0.4× R length; cu-a absent; Rs length 1.4× R length; Cu+M length 0.6× A length; medial vein present, flat medially. Color: body brown to reddish brown; wing membrane brown-tinted, untinted along vein remnants; veins brown.

**Etymology**. The species name, *Loboscelidia fulgens*, refers to the shining integument (Latin, adj).

### Loboscelidia fulva sp. n.

urn:lsid:zoobank.org:act:4719E8B5-6A56-4325-AEE0-4DD9DB50BC1D

http://species-id.net/wiki/Loboscelidia_fulva

Figs 6 27 39

#### Type material.

Holotype male: Thailand: Nan Prov., Doi Phu Kha NP, 19°12'418"N, 101°4'809"E, 1326 m, MT, 15-22 Sept. 2007, Charoen & Nikom, T3217 (QSBG).

#### Diagnosis.

*Loboscelidia fulva* is one of five species with a straight medial vein, including *Loboscelidia meifungae*, *Loboscelidia maculata*, *Loboscelidia ora* and *Loboscelidia defecta*. It can be distinguished from *Loboscelidia ora* and *Loboscelidia maculata* by having notauli, from *Loboscelidia defecta* by having the cu-a vein one-half or more as long as R and Cu+M as long as A, and from *Loboscelidia meifungae* by the rectangular frontal projection, Rs about 3× as long as R and the scutellum coarsely areolate (smooth to longitudinally striate in *Loboscelidia meifungae*).

#### Male description.

**Figures 27–35.**
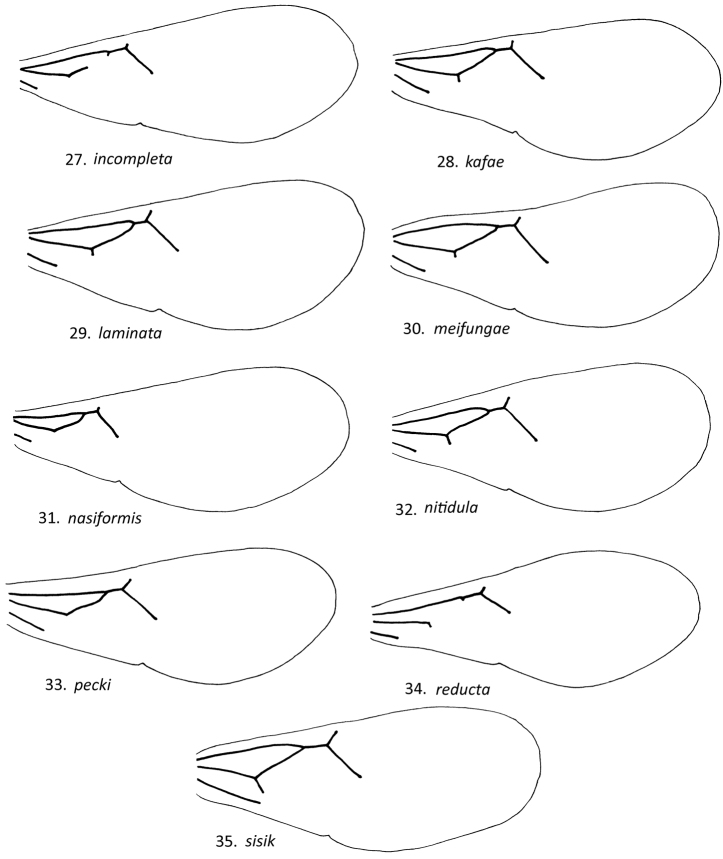
Male *Loboscelidia* forewings.

Body length 2.5 mm; forewing length 3.0 mm. Head (Fig. 6): length 1.6× height in side view; eye asetose; frontal projection rectangular in front view; frons with lateral ridge adjacent to eye margin; vertex without transverse fovea, posterior expansion strongly curved in profile; frons without carina or ridge extending from vertex along inner eye margin; gena without scale-like setae; scape smooth, length 2.7× breadth; flagellomere I length 1.6× breadth; flagellomere II length 1.7× breadth; flagellomere XI length 3.5–4.0× breadth. Mesosoma: pronotal length 0.8× breadth, with lateral carina, as wide as head in dorsal view; scutum with notauli reaching posterior margin; scutellum posteriorly coarsely rugose; metanotum with three medial ridges, impunctate laterally, 0.4× as long as scutellum; mesopleuron with scrobal sulcus; propodeum without transverse dorsal carina; legs (Fig. 39) smooth, polished; forefemoral flange 0.5× femur length, flange maximum width 0.5× width of tubular part of femur; foretibial flange 0.6× tibial length, flange maximum width 0.8× width of tubular part of tibia; midfemoral flange 0.6× femur length, flange maximum width 0.6× of tubular part of femur; midtibial flange 0.8× femur length, flange maximum width 0.7 of tubular part of tibia; hindfemoral flange 0.8× femur length, flange maximum width 0.6× of tubular part of femur; hindtibial flange as long as tibia, flange maximum width 1.2× of tubular part of tibia; hindtibia with two longitudinal carinae on posterior margin; hindcoxa with longitudinal carina on inner medial surface; forewing (Fig. 27) R1 length 0.6× R length; cu-a length 0.6× R length; Rs length 3.1× R length; Cu+M as long as A ; medial vein flat. Color: body dark reddish brown; wing membrane brown-tinted, untinted along vein remnants.

#### Female.

Unknown.

**Figures 36–46.**
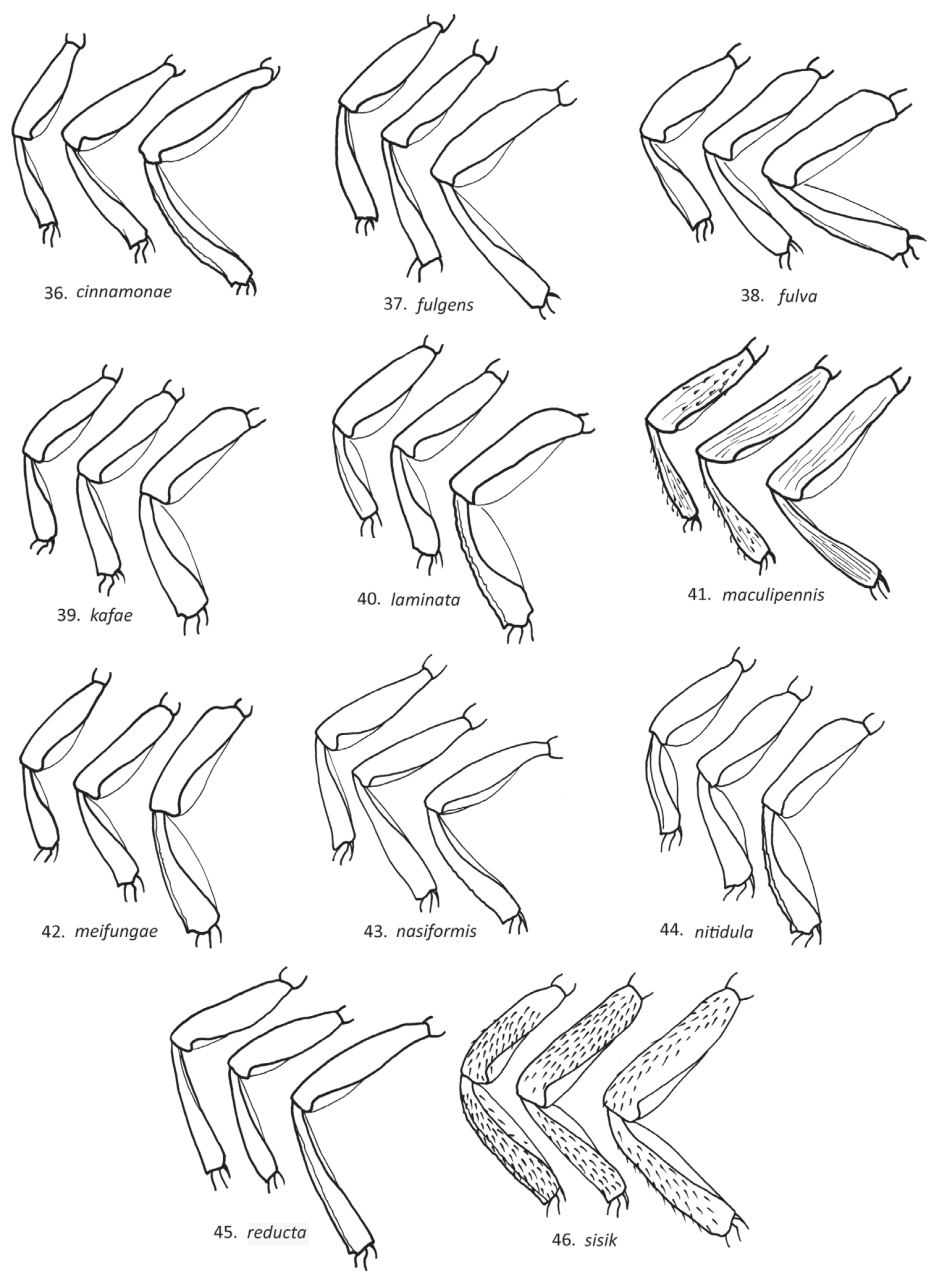
Lateral view of male *Loboscelidia* fore (a), mid (b) and hind (c) legs.

#### Etymology.

The species name, *Loboscelidia fulva*, refers to the brown body color (Latin, *f*.).

### Loboscelidia guangxiensis

Xu

http://species-id.net/wiki/Loboscelidia_guangxiensis

Loboscelidia guangxiensis Xu et al. 2006: 208. Holotype male; China: Guangxi Prov., Jiuwandashan (ZFCL).

#### Material studied.

None; published distribution: China: Guangxi, Guangdong.

#### Diagnosis.

This is one of five species, including *Loboscelidia incompleta*, *Loboscelidia bakeri*, *Loboscelidia reducta* and *Loboscelidia fulgens*, which have the medial vein partial or absent and cu-a less than 0.2× R or absent. It can be distinguished from these species by Rs more than twice as long as R and R1 more than 0.5× as long as R, flagellomeres I and II twice as long as broad, flagellomere XI less than 3× as long as broad and the hindtibial flange less than half as wide as the tubular part of the tibia.

### Loboscelidia halimunensis

Kojima

http://species-id.net/wiki/Loboscelidia_halimunensis

Loboscelidia halimunensis Kojima (in Kojima and Ubaidillah), 2003: 203. Holotype male; Indonesia: West Java, Gunung Halimun National Park, Cikaniki (MZB, lost?).

#### Material studied.

None

**Diagnosis.** This is another of the species with a flat medial vein. *Loboscelidia halimunensis* and *Loboscelidia defecta* both lack a cu-a vein. The two species can be separated by the longer Rs vein in *Loboscelidia halimunensis* (more than 2× as long as R, versus less than 2× in *Loboscelidia defecta*) and pronotum as long as broad or broader (longer than broad in *Loboscelidia defecta*). Despite contacting the authors the type could not be located.

### Loboscelidia incompleta sp. n.

urn:lsid:zoobank.org:act:0C00BA0E-657A-4E11-B707-BD33618B892B

http://species-id.net/wiki/Loboscelidia_incompleta

Figures 8 28

#### Type material.

Holotype male: India: Tamil Nadu, Nilgiri Hills, v/1961, P. S. Nathan (CNC).

#### Diagnosis.

The most distinctive and unique feature of this species is the medially incomplete medial vein. Among the species that lack a medial vein entirely, including *Loboscelidia bakeri*, *Loboscelidia fulgens*, *Loboscelidia reducta* and *Loboscelidia guangxiensis*, *Loboscelidia incompleta* can be distinguished by the Rs vein twice as long as R (1.5× or less in the other species). It does share the fore and midtibial flanges lacking as in *Loboscelidia reducta*.

#### Male description.

Body length 2.5 mm; forewing length 3 mm. Head (Fig. 8): length 2× height in side view; eye asetose; frontal projection rectangular in front view; frons with lateral ridge adjacent to eye margin; vertex without transverse fovea, posterior expansion strongly curved in profile; frons without carina or ridge extending from vertex along inner eye margin; gena without scale-like setae; scape longitudinally striate, length 4× breadth; flagellomere I length 2.4× breadth; flagellomere II length 2.2× breadth; flagellomere XI length 3.2× breadth. Mesosoma: pronotal length 1.1× breadth, with lateral carina, nearly as broad as head; scutum with notauli reaching posterior margin; scutellum and metanotum smooth, polished, impunctate; metanotum one-third as long as scutellum propodeum without transverse dorsal carina; mesopleuron without scrobal sulcus; legs polished; forefemoral flange 0.2× femur length, flange maximum width 0.9× width of tubular part of femur; foretibial flange 0.6× femur length, flange maximum width 0.3× width of tubular part of tibia; midfemur without flange; midtibial flange 0.7× tibia length, flange maximum width 0.3× width of tubular part of tibia; hindfemoral flange 0.3× femur length, flange maximum width 0.7× width of tubular part of femur; hindtibial flange 0.7× as long as tibia, flange maximum width 0.5× width of tubular part of tibia; hindtibia with two longitudinal carinae on posterior margin; hindcoxa with/without longitudinal carina on inner medial surface; forewing (Fig. 28) R1 length 0.3× R length; cu-a length absent; Rs length 2.2× R length; Cu+M 0.5× as long as A; medial vein submedially curved, incomplete medially. Color: reddish brown; wing membrane brown-tinted, paler along vein remnants, veins brown.

#### Etymology. 

The name refers to the medially interrupted medial vein of the forewing (Latin)

### Loboscelidia indica

Kimsey

http://species-id.net/wiki/Loboscelidia_indica

Loboscelidia indica Kimsey 1988: 69. Holotype male; India: Nilgiri Hills (CNC).

#### Material studied.

India: Nilgiri; only the holotype was seen.

#### Diagnosis.

*Loboscelidia indica* is one of two species described from India, including *Loboscelidia incompleta*. It is also one of the dozen or so species with a scrobal sulcus and rectangular frontal projection. It can be distinguished from them by the combination of the Rs less than 3× as long as R, A as long or longer than Cu+M, scape less than 3× as long as broad, flagellomeres I and II twice or more as long as broad, and fore, mid and hindtibial flanges 1.5× or more as wide as the tibiae.

### Loboscelidia inermis

Kieffer

http://species-id.net/wiki/Loboscelidia_inermis

Loboscelidia inermis Kieffer 1916: 15. Syntype females (males?); Philippines: Mindanao, Butuan (MNHN, lost?).

#### Material studied.

No reliably identified specimens have been seen. However, according to Kieffer's (1916) illustration *Loboscelidia inermis* has a well-developed cu-a vein, unlike *Loboscelidia defecta*, which lacks cu-a, or cu-a is represented by a very short stub.

### Loboscelidia kafae sp. n.

urn:lsid:zoobank.org:act:09492B77-D0B2-401F-94AA-863039EF6EA8

http://species-id.net/wiki/Loboscelidia_kafae

Figures 9 29 40

#### Type material.

Holotype male: Thailand: Chiang Mai Pr., Doi Phahompok NP, Mae Fang Hot spring, 569m, 19°57.961'N, 99°09.355'E, Malaise trap, 7–14/iv/2008, K. Seesom, T6085 (QSBG).

Paratypes (52 males): 2 males: same data as holotype; 1 male: 14-21/ix.2007, P. Wongchai, T6168; 2 males:, 7–14/viii/2007, P. Wongchai, T6144, 6111; 1 male: Doi Phaluang, 1449 m, 20°1'06N, 99°09.581'E, 21–28/ix/2007, P. Wongchai, T6165; 1 male: 28/iv-7/v/2008, K. Seesom, T6084; 1 male: Doi Chiangdao NP, 19°24.278'N, 98°55.311'E, 491 m, 18–25/ix/2007, Jugsu & Watwanich, T5696; 1 male: Doi Chiangdao NP, 19°24.419'N, 98°55.237'E, 549 m, MT, 21–28/viii/2007, Jugsu & Watwanich, T5676; 1 male: Doi Chiangdao NP, Pha Tang, 19°24.978'N, 98°54.886'E, 526 m, Malaise trap, 4–11/ix/2007, Jugsu & Watwanich, T5682; 1 male: Doi Chiangdao NP, 549 m, 19°42.419'N, 98°55.237'E, Malaise trap, 10–17/xii/2007, Jugsu & Watwanich, T5723; 1 male: Haui Na Lao, 500 m, 19°24.731'N, 98°55.315'E, Malaise trap, 15–21/v/2008, Jugsu & Watwanich, T5817; 1 male: Huai Nam Dang NP, 19°18.803'N, 98°36.396'E, Malaise trap, 21–28/ix/2007, Anuchart & Thawatchai, T5507; 1 male: Thung Buatong viewpoint, 19°17.6'N, 93°36.0'E Malaise trap, Anuchart & Thawatchai, 14–21/viii/2007, T5472; 1 male: **Chiang Pr.**, Huai Nam Dang NP, Thung Buatong, 19°17.056'N, 98°36.029'E, Malaise trap, 21–28/viii/2007, Anuchart & Thawatchai, T5471; 1 male: Doi Chiangdao NP, 19°24.419'N, 98°55.237'E, 549 m, malaise trap, 14–21/viii/2007, Jugsu & Watwanich, T5673; 1 male: **Kamphaeng Pr.**, Phet Mae Wong NP, 306 m, 16°02.233'N, 99°13.096'E, pan trap, 9–10/viii/2007, Srilopien & Phumirate, T3769; 1 male: **Lampang Pr.**, Chae Son NP, 18°49.894'N, 99°28.354'E, 467 m, Malaise trap, 1–7/v/2008, Kwannui & Sukpeng, T5309; 1 male: 21–30/v/2008, T5305; 1 male: Chae Son NP, Doi Laan, 18°51.815'N, 99°22.122'E, 1413 m, Malaise trap, 9–15/v/2008, Kwannui & Sukpeng, T5292; 1 male: **Kanchanaburi Pr.**, Khuean Srinagarinda NP, 14°38.123'N, 98°59.657'E, Malaise trap, Somboon & Daorueng, T3462; 1 male: 7–14/v/2009, T4747; 1 male: 201 m, 23–30/iv/2009, T4744; 1 male: 13–20/xi/2008, Somboon & Daorueng, T4423; 1 male: 6–13/xi/2008, Somboon & Daorueng, T4420; 1 male; 14°38.312'N, 98°59.643'E, 210 m, Malaise trap, Somboon & Daorueng, T3465; 1 male: Huay Mae Kamint, 14°38.441'N, 98°58.889'E, 240 m, Malaise trap, 7–14/v/2009, Somboon & Daorueng, T4740; 1 male: **Nakhon Si Thammarat Pr.**, Namtok Yong NP, 8°10.434'N, 99°44.508'E, Malaise trap, 8–15/vii/2008, 80 m, U. prai, KT3083; .1 male: 8°14.262'N, 99°48.289'E, Malaise trap, 21–28/vii/2008, 966m, Palboon, T3108; 1 male: 8°16.959'N, 99°39.149'E, Malaise trap, 22–29/vii/2008; 1 male: road to Khao Mhen, 150 m from Nern466, 8°16.959'N, 99°39.149'E, 499 m, Malaise trap, 8–15/vi/2008, S. Samnaokan, T3095; 1 male: **Chaiyaphum Pr.**, Tat Tone NP, 16°0.792'N, 101°58.472'E, Malaise trap, 19–26/v/2007, Jaruphan & Budsawong, 648 m, 2575; 2 males: **Petchaburi Pr.**, Kaeng Krachan NP, 12°47.831'N, 99°27.369'E, Malaise trap, 970 m, 8–15/viii/2008, Sirichai & Chusak, T4346; 1 male: 12°47.963'N, 99°27.188'E, Malaise trap, 5–12/ix/2008, Sirichai & Prasit, T4375; 1 male: 12°50.177'N, 99°28.098'E, Malaise trap, 735 m, 18–25/i/2009, Sirichai, T4406; 1 male: 12°48.107'N, 99°26.669'E, Malaise trap, 3–10/iv/2009, Sirichai, T4687; 1 male: 12°49.302'N, 99°22.263'E, Malaise trap, 254/iii-3/iv/2009, Sirichai, T4739; 1 male: 12°50.177'N, 99°20.688'E, Malaise trap, 735 m, 25/v-1/vi/2009, Sirichai, T5259; 2 males: Pa La-U waterfall, 12°32.154'N, 99°28.098'E, Malaise trap, 26/ix-3/x/2008, Akaradate & Thongbai, T4518; 1 male: 12°32.154'N, 99°28.098'E, Malaise trap, 4–11/xii/2008, Thongbai, T4553; 1 male: Pa La-U/Huai Palao Forest Unit 3, 12°32.149'N, 99°28.265'E, Malaise trap, 18–25/i/2009, Thongbai, T4566; 1 male: 12°32.149'N, 99°28.265'E, Malaise trap, 4–11/i/2009, Thongbai, T4562; 2 males: **Phetchabun Pr.**, Nam Nao NP, 16°43.695'N, 101°33.797'E, 921 m, Malaise trap, 5–12/v/2007, L. Janteab, T2657; 2 males: 16°43.687'N, 101°33.797'E, 754 m, Malaise trap, 19–26/v/2007, N. Hongyothi, T2662; 1 male: **Mae Hong Son Pr.**, Namtok Mae Surin NP, 228 m, 19°21.593'N, 97°59.254'E, Malaise trap, 11–18/xi/2007, M. Namadkum, T5930; 1 male: 19°20.616'N, 97°59.003'E, Malaise trap, 11–18/xi/2007, 334 m, A. Kamkhun, T5934; 1 male: **Sakon Nakhon Pr.**, Phu Phan NP, 17°03.488'N, 103°58.497'E, Malaise trap, 8–14/vii/2006, S. Tongboonchai, T199; 1 male: **Prachuab Khiri Khan Pr.**, Khao Sam Roi Yot NP, 12°13.417'N, 99°56.153'E, Malaise trap, 31/viii-7/ix/2008, Sorat, Yai & Amnad, T4078; 1 male: Bar Hua Tan Thaeo, 12°13.059'N, 99°58.384'E, Malaise trap, 2–9/xi/2008, Yai & Amnad, T4128; 1 male: **Phitsanulok Pr.**, Thung Salaeng Luang NP, 16°52.046'N, 100°49.067'E, Malaise trap, 501 m, 16–23/iv/2007, Pongpitak, T5207 (BME, QSBG).

Additional non-type specimens were seen from Laos (Phongsaly Prov., Ban Sano Mai) (22 males, CNC, BME); Vientiane Prov., Ban Van Eue (1 male, BPBM); Malaysia: Malaya, 13 mi e Gombak (1 male, UCR); Sarawak: Gunung Mulu NP (1 male, ROM) and Borneo: West Kalimantan Gunung Palung Nat Pk. (14 males, BME, ROM) E. Kalimantan: Kac. Plujungan, Kayan Metarang Nat. Res. (1 male, ROM); Viet Nam: Tuyen Quang Prov., Na Hang Res. (2 males, CNC); Thailand: Phitsanulok Pr., Thyng Salaeng Luang (1 males, BME, QSBG); Kanchanaburi: Khuean Srinagarinda NP (1 male, QSBG); Suphanburi Pro., Pu Toei NP (1 male, QSBG).

#### Diagnosis.

L.* kafae* is one of the many species that have a submedially curved medial vein. Males have a short flagellomere I (less than twice as long as broad), which is also found in *Loboscelidia pasohana* and *Loboscelidia laminata*. It can be distinguished from these two species by flagellomere XI 4× as long as broad (shorter in the other species), the fore and midtibial flanges as broad as the tibiae and the hindtibial flange twice as broad (narrower in various combinations in the other species).

#### Male description.

Body length 2.0–2.5 mm; forewing length 2.5–3.0 mm. Head (Fig. 9): length 1.9× height in side view; eye asetose; frontal projection rectangular in front view; frons smooth; vertex without transverse fovea, posterior expansion convex in profile; frons with low ridge extending from vertex along inner eye margin; gena without scale-like setae; scape smooth, length 3× breadth; flagellomere I length 1.6× breadth; flagellomere II length 2× breadth; flagellomere XI length 4.5× breadth. Mesosoma: pronotal length 0.8× breadth, with sharp lateral fold; scutum with notauli reaching posterior margin; scutellum with fine dense striae sublaterally; metanotum with medial ridge, densely, finely striate on either side, one-third as long as scutellum; mesopleuron with scrobal sulcus; propodeum without transverse dorsal carina; legs (Fig. 40) smooth, polished; forefemoral flange 0.7× femur length, flange maximum width 0.9× width of tubular part of femur; foretibial flange 0.7× femur length, flange maximum width 1.2× width of tubular part of tibia; midfemoral flange 0.8× femur length, flange as wide as tubular part of femur; midtibial flange 0.7× tibial length, flange as wide as tubular part of tibia; hindfemoral flange 0.9× femur length, flange maximum width as wide as tubular part of femur; hindtibial flange as long as femur, flange maximum width 2× width of tubular part of tibia; hindtibia with two longitudinal carinae on posterior margin; hindcoxa without longitudinal carina on inner medial surface; forewing (Fig. 29) R1 length 0.5× R length; cu-a 0.5× R length; Rs length 2.6× R length; Cu+M length 0.5× A length; medial vein submedially curved. Color: body brown; wing membrane lightly brown-tinted along veins and vein remnants, veins brown.

#### Female.

Unknown.

#### Etymology.

The species name refers to the coffee brown coloration (Thai for coffee, noun).

### Loboscelidia laminata sp. n.

urn:lsid:zoobank.org:act:30E0EEB4-A91F-49CE-9CEA-E86A911F785A

http://species-id.net/wiki/Loboscelidia_laminata

Figures 10 30 41

#### Type material.

Holotype male: Viet Nam: Tuyen Quang Prov., 360 m, Na Hang Reserve, 16–20 May 1997, FIT, S. B. Peck, 97-10 (CNC). Paratypes (17 males): 6 males, same data as holotype; 6 males, 20-24 May 1997, rainforest, FIT 97-13; 5 males, 97-12 (BME, CNC).

#### Diagnosis.

*Loboscelidia laminata* most closely resembles *Loboscelidia kafae* as discussed under that species. However, *Loboscelidia laminata* can be distinguished by flagellomere II less than twice as long as broad, flagellomere XI less than 3.5× as long as broad, and the fore and midtibial flanges narrower than the respective tibiae.

#### Male description.

Body length 2.0–2.5 mm; forewing length 2.5–3.0 mm. Head (Fig. 10): length 1.8× height in side view; eye asetose; frontal projection rectangular in front view; frons with lateral ridge adjacent to eye margin; vertex without transverse fovea, posterior expansion shallowly curved in profile; frons with low ridge extending from vertex along inner eye margin; gena without scale-like setae; scape smooth, length 2.6× breadth; flagellomere I length 1.7× breadth; flagellomere II length 1.8× breadth; flagellomere XI length 4× breadth. Mesosoma: pronotal length 0.8× breadth, with/out lateral carina, nearly as wide as head in dorsal view; scutum with notauli reaching posterior margin; scutellum with fine dense striae; metanotum with three medial ridges, impunctate laterally; mesopleuron with scrobal sulcus; propodeum without transverse dorsal carina; legs (Fig. 41) coarsely/smooth, polished; forefemoral flange 0.6× femur length, flange maximum width 0.8× width of tubular part of femur; foretibial flange 0.6× femur length, flange maximum width 0.7× width of tubular part of tibia; midfemoral flange 0.6× femur length, flange maximum width 0.8× width of tubular part of femur; midtibial flange 0.8× femur length, flange maximum width 0.4× width of tubular part of tibia; hindfemoral flange 0.8× femur length, flange maximum width 0.9× width of tubular part of femur; hindtibial flange 0.9× as long as tibia, flange maximum width 1.1× width of tubular part of tibia; hindtibia with two longitudinal carinae on posterior margin; hindcoxa with longitudinal carina on inner medial surface; forewing (Fig. 30) R1 length 0.8× R length; cu-a length 0.5× R length; Rs length 3.2× R length; Cu+M 0.5× as long as A ; medial vein submedially curved. Color: dark brown to yellowish brown; wing membrane brown-tinted, untinted along vein remnants; veins brown.

#### Etymology.

The name refers to the large lamellae or flanges on the legs (Latin).

### Loboscelidia laotiana

Kimsey

http://species-id.net/wiki/Loboscelidia_laotiana

Figure 19

Loboscelidia laotiana Kimsey 1988: 71. Holotype male; Laos: Vientiane Prov., Ban Van Eue (BPBM).

#### Material studied.

Laos: Vientiane Prov, Ban Van Eue (2 males, BPBM, BME); Viet Nam: Fyan (1 male, BME); Malaysia: Sabah: Kinabalu Nat. Pk. (3 males, USNM); Indonesia: Sumatra, Aceh: Mt. Leuser Nat. Pk., Ketambe Res. Sta (1 male, ROM); 7 specimens were seen including the holotype.

#### Diagnosis.

*Loboscelidia laotiana* is one of the species with a scrobal sulcus and a triangular frontal projection (Fig. 19). It can be distinguished from the others by the combination of the Rs 3× or more as long as R, scape striate and more than 3× as long as broad, flagellomeres I and II less than twice as long as broad, flagellomere XI less than 3× as long as broad, fore and midfemoral flanges as wide as the tubular part of the respective femora and the hindtibial flange twice as wide as the tubular part of the tibia.

### Loboscelidia levigata

Yao, Liu & Xu

http://species-id.net/wiki/Loboscelidia_levigata

Loboscelidia levigata Yao et al. 2010: 528. Holotype male; China: Guangdong Prov., Chebaling National Nature Reserve (SCAC).

#### Material studied.

None.

#### Diagnosis.

*Loboscelidia levigata* is one of three species described from southeastern China, including *Loboscelidia sinensis* and *Loboscelidia striolata*. It can be distinguished from these by the rectangular frontal projection, and in males R1 as long as R (shorter in *Loboscelidia sinensis* and *Loboscelidia striolata*) and Rs 3× as long as R, as opposed to 2.5× or shorter in *Loboscelidia sinensis* and *Loboscelidia striolata*. It can be distinguished from other *Loboscelidia* species by R1 reaching R at a right angle.

### Loboscelidia maai

(Lin)

http://species-id.net/wiki/Loboscelidia_maai

Scelidoloba maai Lin 1964: 238. Holotype female (not male); Taiwan: Paomingszu, 2 km s Keelung City (NMNS).
Loboscelidia artigena Lin 1964: 243. Holotype male; Taiwan: Paomingzu, 2 km s Keelung City (NMNS). Possible synonymy with *Loboscelidia maai* suggested by Day (1979). New synonymy herein.
Loboscelidia latigena Lin 1964: 241. Holotype male; Taiwan: Tsaoshan, 20 km nw Taipei city (NMNS). Synonymized by Kimsey and Bohart 1991.

#### Material studied.

None.

#### Diagnosis.

This is one of four species, including *Loboscelidia brunnea*, *Loboscelidia maculipennis* and *Loboscelidia nitidula*, with the cervical expansion of the vertex flat in profile. *Loboscelidia maai* males can be distinguished from these species by having the scape less than 2.5× as long as broad, the presence of a scrobal sulcus, and the tibial flanges wider than the tubular part of the respective tibiae.

### Loboscelidia maculata

Kimsey

http://species-id.net/wiki/Loboscelidia_maculata

Loboscelidia maculata Kimsey 1988: 72. Holotype male; Australia: Queensland, 7 km sw Bellenden (ANIC).

#### Material studied.

Australia: Queensland: 7 km sw Bellenden (1 male, ANIC); Mossman Gorge (2 males, CNC); 3 specimens were seen including the holotype.

#### Diagnosis.

This is one of the five species with a medially flat medial vein as discussed under *Loboscelidia defecta*. Of these, only *Loboscelidia defecta* and *Loboscelidia ora* have been described from Australia. *Loboscelidia maculata* can be distinguished from *Loboscelidia defecta* by the lack of notauli (shared with *Loboscelidia ora*), and the fore and hindtibial flanges twice as wide as the tubular part of the respective tibiae (narrower in *Loboscelidia defecta* and *Loboscelidia ora*).

### Loboscelidia maculipennis

Fouts

http://species-id.net/wiki/Loboscelidia_maculipennis

Figures 11 17, 20 41

Loboscelidia maculipennis Fouts 1922: 625. Holotype male (not female); Borneo: Sandakan (USNM).
Loboscelidia carinata Fouts 1922: 626. Holotype male (not female); Singapore (USNM). Synonymized by Day (1979).

#### Material studied.

Singapore: coll. Baker (1 make, BME), Sungei Bulch (1 male, BME); Indonesia: W. Kalimantan: Gunung Palung Nat. Pk. (6 males, ROM, BME); E. Kalimantan: Kac. Pujungan, Kayen-Mentarang Nat. Res (1 male, ROM), Sumatra: Aceh, Mt. Leuser (1 male, ROM); 12 males were seen including the holotypes of *Loboscelidia maculipennis* and *Loboscelidia carinata*.

**Diagnosis.** This is one of four species with a strongly flattened cervical expansion (Fig. 11) as discussed under *Loboscelidia brunnea*. *Loboscelidia maculipennis* males can be distinguished from the other three by cu-a as long as R, Rs vein 4× or longer than R, leg integument smooth (Fig. 41), and hindtibial posterior margin with 2 parallel carinae.

### Loboscelidia meifungae sp. n.

urn:lsid:zoobank.org:act:D05A300F-E49B-476E-98D4-970C53404F6B

http://species-id.net/wiki/Loboscelidia_meifungae

Figures 12 30 42

#### Type material.

Holotype male: Borneo: Sarawak, sw Gunung Buda, 64 km s Linbang, 4°13'N, 114°56'E, 8–15 Nov. 1996, MT, Heydon & Fung (BME). Paratypes (44): 10 males, same data as holotype; 10 males: 16–28 Nov. 1996; 11 males: 22-28 Nov. 1996, MT, Heydon & Fung; 1 male: November 1996, Heydon & Fung; 1 male: 18 Nov. 1996, Heydon & Fung; 1 male: 23 Nov. 1996; Heydon & Fung; 1 male: Buda Camp, sw Gunung Buda, 64 km s Linbang, 4°11'N, 114°56'E, 4 Nov. 1996, MT, Heydon & Fung; 4 males: Malaysia: Sabah, Kinabalu NP, 800m, Poring Hot Springs Langanan Creek, 22/viii/1988, A. Smetana, B-138; 1 male: Poring Hot Springs, 520 m, 9/v/1987, A. Smetana; 1 male: 480–510 m, 30.viii/1988, A. Smetana, B163; 1 male: 510 m, 13/v/1987; 1 male: Kipungit Creek, 550 m, 26/viii/1988, A. Smetana; 1 male: Liwagu River Trail, 1550 m, 12/viii/1988, A. Smetana, B107 (BME, CNC).

#### Diagnosis.

This species belongs in the group of species having a flat medial vein and notauli, including *Loboscelidia defecta* and *Loboscelidia fulva*. It can be distinguished from other members of the group by the triangular frontal projection, presence of a scrobal sulcus, cu-a present (shared with *Loboscelidia fulva*) and midtibial flange absent.

#### Male description.

Body length 2.0–4.0 mm; forewing length 2.5–4.5 mm. Head (Fig. 12): length 1.8–2.0× height in side view; eye asetose; frontal projection triangular in front view; frons smooth to microstriate; vertex without transverse fovea, posterior expansion strongly curved in profile; frons without discrete carina or ridge extending from vertex along inner eye margin; gena without scale-like setae; scape with some striae, length 2.1–2.5× breadth; flagellomeres I and II length twice breadth; flagellomere XI length 4× breadth. Mesosoma: pronotal length 0.7–0.8× breadth, with lateral carina; scutum with notauli reaching posterior margin; scutellum with sublateral carina, with fine dense striae laterally; scrobal sulcus represented by series of pits; metanotum with medial ridge, impunctate laterally; propodeum without transverse dorsal carina; legs (Fig. 42) smooth, polished; forefemoral flange 0.5–0.7× femur length, flange maximum width 0.8-1.0× width of tubular part of femur; foretibial flange 0.6–0.9× femur length, flange maximum width 1.0–1.5 x width of tubular part of tibia; midfemoral flange 0.7–0.9× femur length, flange maximum width as wide as tubular part of femur; midtibial flange 0.7× femur length, flange maximum width 1.2× width of tubular part of tibia; hindfemoral flange 0.9× femur length, flange maximum width as wide as tubular part of femur; hindtibial flange 0.9× femur length, flange maximum width 1.7× width of tubular part of tibia; hindtibia with two longitudinal carinae on posterior margin; hindcoxa without longitudinal carina on inner medial surface; forewing (Fig. 30) R1 length 0.5–0.7× R length; cu-a length 0.4–0.5× R length; Rs length 2.5–3.0× R length; Cu+M length 0.7–0.9× A length; medial vein submedially curved. Color: body brown to reddish brown; wing membrane brown-tinted, paler along vein remnants.

#### Female.

Unknown.

#### Etymology.

This species is named after Mei Lin “Stella” Fung one of the collectors.

### Loboscelidia nasiformis sp. n.

urn:lsid:zoobank.org:act:BD5AC828-B80E-45BB-8835-1D6EAFCFDAA4

http://species-id.net/wiki/Loboscelidia_nasiformis

Figures 13 31 43

#### Type material.

Holotype male: Thailand: Petchaburi Prov., Kaeng Krachan NP, Pa La-U/Huai Palao Forest Unit 3, 12°32'149"N, 99°28'265"E, Malaise trap, 4-11/i2009, Thongbai, T4562 (QSBG).

#### Diagnosis.

The most distinctive and unusual feature of this species is the greatly elongate and nose-like frontal projection, which makes the head nearly 3× as long as broad in lateral view. Otherwise, *Loboscelidia nasiformis* is closest to *Loboscelidia cinnamonea*, with an arched medial vein, rectangular frontal projection (albeit greatly elongate in *Loboscelidia nasiformis*), complete notauli, cu-a reduced to a tiny stub or absent, and no scrobal sulcus. Other than the elongate frontal projection, *Loboscelidia nasiformis* can be separated from *Loboscelidia cinnamonea* by the presence of fore and midtibial flanges (absent in *Loboscelidia cinnamonea*).

#### Male description. 

Body length 2 mm; forewing length 2.5 mm. Head (Fig. 13): length 2.9× height in side view; eye asetose; frontal projection nasiform; frons smooth; vertex without transverse fovea, posterior expansion strongly curved in profile; frons without carina or ridge extending from vertex along inner eye margin; gena without scale-like setae; scape smooth, without striae, length 3.7× breadth; flagellomeres I and II length 2.2× breadth; flagellomere XI length 3.6× breadth. Mesosoma: pronotal length 0.9× breadth, without lateral carina; scutum with notauli reaching posterior margin; scutellum without sublateral carina, smooth laterally; metanotum without medial ridge, impunctate laterally; propodeum without transverse dorsal carina; legs (Fig. 43) smooth, polished; forefemoral flange 0.5× femur length, flange as wide as tubular part of femur; foretibial flange 0.6× femur length, flange maximum width 0.4× width of tubular part of tibia; midfemoral flange 0.5× femur length, flange maximum width 0.4× width of tubular part of femur; midtibial flange 0.6× femur length, flange maximum width 0.6× width of tubular part of tibia; hindfemoral flange 0.8× femur length, flange maximum width 0.5× width of tubular part of femur; hindtibial flange 0.8× femur length, flange maximum width 0.6× width of tubular part of tibia; hindtibia with two longitudinal carinae on posterior margin; hindcoxa without longitudinal carina on inner medial surface; forewing (Fig. 31) R1 length 0.3× R length; cu-a absent; Rs length 2.6× R length; Cu+M length 0.5× A length; medial vein submedially curved. Color: body brown to reddish brown; wing membrane brown-tinted, paler along vein remnants.

#### Etymology.

The species is named for the long, nose-like frontal projection (Latin)

### Loboscelidia nigra

Fouts

http://species-id.net/wiki/Loboscelidia_nigra

Loboscelidia nigra Fouts 1922: 621. Syntype males (not female); Philippines: Mindanao, Dapitan, Basilan (USNM).

#### Material studied.

Philippines: Mindanao; only the two syntypes were seen.

#### Diagnosis.

As discussed under *Loboscelidia castanea* and *Loboscelidia collaris*, *Loboscelidia nigra* is one of seven species with a triangular frontal projection, complete scrobal sulcus and complete notauli. Dimensions of the antennal articles will separate *Loboscelidia nigra* from these species; the scape is less than 3× as long as broad, flagellomeres I and II are 2.5× as long as broad or longer and flagellomere XI is 4.5× as long as broad.

### Loboscelidia nigricephala

Kimsey

http://species-id.net/wiki/Loboscelidia_nigricephala

Loboscelidia nigricephala Kimsey 1988: 72. Holotype male; Australia: Queensland, 21 km s Atherton (QDPI).

#### Material studied.

Australia: Queensland: Mt. Lewis (1 male, CNC); 21 km s Atherton (1 male, QDPI); Hugh Nelson Range, s Atherton (1 male, BME); 3 males were seen, including the holotype.

#### Diagnosis.

This is one of five species, including *Loboscelidia cervix*, *Loboscelidia novoguineana*, *Loboscelidia ora* and *Loboscelidia parva*, where the frontal projection is broadly flattened and nearly linear in front view. It can be distinguished from these species by the arched medial vein, cu-a as long as or longer than R, foretibial flange as wide as tubular part of tibia, and the mid and hindtibial flanges 1.5× as wide as tubular part of the tibiae or wider.

### Loboscelidia nigricornis

Fouts

http://species-id.net/wiki/Loboscelidia_nigricornis

Loboscelidia nigricornis Fouts 1925: 517. Holotype male; Philippines: Mindanao, Surigao (USNM).

#### Material studied.

Philippines: Mindanao I., Agusan, Esperanza Bagugan, Matibog Creek (1 male, BPBM); 6 specimens were seen including the otype series.

#### Diagnosis.

This is one of several species with a flat medial vein and rectangular frontal projection. In males, the absence of cu-a and the scape more than 3.3× as long as broad are characteristics *Loboscelidia nigricornis* shares with *Loboscelidia halimunensis*. *Loboscelidia defecta* can be distinguished from *Loboscelidia halimunensis* by Rs less than twice as long as broad (longer in *Loboscelidia halimunensis*) and longer pronotum (1.2× as long as broad, versus as long as broad or broader in *Loboscelidia halimunensis*).

### Loboscelidia nitidula sp. n.

urn:lsid:zoobank.org:act:B9D54654-B75A-4659-A223-901AFCCDB5BD

http://species-id.net/wiki/Loboscelidia_nitidula

Figures 32 44

#### Type material.

Holotype male: Thailand, Petchaburi Prov., Nam Nao NP, 16°43'687"N, 101°33'754"E, 924 m, MT, 5-12/v/2007, N. Hongyothi, T2656 (QSBG). Paratypes (21 males): 1 male: Kaeng Krachan NP, 16/road/stream, 12°48'189"N, 99°26'62"E, MT, 11–18/iii/2009, Sirichai & Prasit, T4685; 1 male: 12°50'177"N, 99°20'688"E, MT, 735 m, 27/xi-4/xii/2008, Sirichai, T4395; 2 males: Chang Mai Prov., Doi Inthanon NP, 7–12/v/1990, E. Fuller, MT; 1 male: Chiangdao NP, Huai Na Lao, 19°24'731"N, 98°55'315"E, 500 m, YPT, 6-7/v/2008, Jugsu & Watwanich, T5808; 1 male: Sakon Nakhon Prov., Phu Phan NP, 14/vii/2006, 17°03'543"N, 103°58'452"E, MT 8-W, Kongnara, T197; 2 males: 17°03'543"N, 103°58'452"E, MT, 15–21/vii/2006, MT, S. Tongboonchai, T200; 3 males: 17°03'488"N, 103°58'497"E, MT, 15–21/vii/2006, MT, S. Tongboonchai, T205; 1 male: Nong Bua Prov., Lam Poo Phu Kao Phu, Phan Kham Nat. Pk., 16°49'N, 102°37'E, 208 m, 27/vii-2/viii/2006, MT, R. Singhatip, T85; 1 male: Nakhon Si Prov., Thammarat, Namtok Yong NP, 17°10'434"N, 99°44'508"E, 80 m, MT, 16–23/viii/2008, K. Uprai, T3548; 2 males: Kanchanaburi Prov., Khuean Srinagarindra NP, Huay Mae Kamint, 14°38'312"N, 98°5'643"E, 210 m, MT, 13–20/xi/2008, Somboon & Daorueng, T4424/4423; 1 male: Erawan NP, 100 m, 5/vii/1990, J. Heraty, 90/115; 1 male: Loei Prov., Phu Kradeung NP, 16°49'01"N, 101°47'62"E, 276 m, MT, 14–21/v/2008, T. Phatai, T5011; 1 male: Trang Prov., Nayong, 7 m, 20/ii/2005, 7°33'04"N, 99°49'37"E, MT, D. Lohman; 1 male: Khao Chong Mt. 75 m, 28/iv-2/v/2005, 7°33'38"N, 99°47'369"E, MT; 1 male: Khao Chong Mt. 75 m, x/2005, 7°33'38"N, 99°47'369"E, MT; 1 male: near Nam Tock Ton Prov., Khoa Chong Mt., 140 m, ii/2005, 7°32'15"N, 99°47'36"E, MT, D. Lohman (QSBG, BME, CNC).

#### Diagnosis.

Four *Loboscelidia* species have a flattened cervical extension, including *brunnea, maculipennis* and *Loboscelidia nitidula*. Of these four *Loboscelidia nitidula* can be distinguished by presence of a scrobal sulcus, a medial metanotal ridge and a large foretibial flange (flange absent in the other species).

#### Male description.

Body length 2.0-2.5 mm; forewing length 2.5-3.0 mm. Head: length 1.6× height in side view; eye asetose; frontal projection rectangular in front view; frons smooth, not microstriate; vertex without transverse fovea, posterior expansion convex in profile; frons with ridge extending from vertex along inner eye margin; gena without scale-like setae; scape striate, length 2.7 breadth; flagellomere I length 1.7× breadth; flagellomere II length 1.8× breadth; flagellomere XI length 5× breadth. Mesosoma: pronotal length 0.8× breadth, with fold between dorsal and lateral surfaces, as wide as head width in dorsal view; scutum with notauli reaching posterior margin; scutellum with fine dense striae laterally; metanotum with medial longitudinal striae, impunctate laterally, 0.5× as long as scutellum; mesopleuron with scrobal sulcus; propodeum without transverse dorsal carina; legs (Fig. 44) smooth polished; forefemoral flange 0.7× femur length, flange maximum width 0.8× width of tubular part of femur; foretibial flange 0.9× femur length, flange maximum width as wide as tubular part of tibia; midfemoral flange 0.7× femur length, flange maximum width 0.7× width of tubular part of femur; midtibial flange 0.8× femur length, flange maximum width 1.2× width of tubular part of tibia; hindfemoral flange as long as femur, flange maximum width 1.2× width of tubular part of femur; hindtibial flange as long as femur, flange maximum width 1.6× width of tubular part of tibia; hindtibia with two longitudinal carinae on posterior margin; hindcoxa without longitudinal carina on inner medial surface; forewing (Fig. 32) R1 length 0.7× R length; cu-a length 0.6× R length; Rs length 2.9× R length; Cu+M length 0.6× A length; medial vein submedially curved. Color: body brown to reddish brown; wing membrane brown-tinted, darkest medially, lightest along vein remnants.

#### Etymology.

The species name, *Loboscelidia nitidula*, is Latin for shiny/polished (f.).

### Loboscelidia nixoni

Day

http://species-id.net/wiki/Loboscelidia_nixoni

Laccomerista rufescens Cameron 1910a: 23. Holotype male; Borneo: Kuching (BMNH). Nec Westwood 1874.
Loboscelidia nixoni Day 1978: 29. Replacement name for *Loboscelidia rufescens* (Cameron 1910).

#### Material studied.

Borneo; only the holotype of *Loboscelidia rufescens* (Cameron) was seen.

#### Diagnosis.

*Loboscelidia nixoni* is another of the species characterized by having a curved medial vein, rectangular frontal projection, and no scrobal sulcus, as discussed under *Loboscelidia philippinensis*. In this group *Loboscelidia nixoni* differs from *Loboscelidia nasiformis* and *Loboscelidia cinnamonea* in having cu-a well-developed and half as long as R. It can be separated from *Loboscelidia philippinensis*, and *Loboscelidia levigata* by the combination of the scape and flagellomere XI less than twice as long as broad, flagellomeres I and II less than 1.7× as long as broad and hindtibial flange less than 0.7× as wide as tubular part of tibia.

### Loboscelidia novoguineana

Kimsey

http://species-id.net/wiki/Loboscelidia_novoguineana

Loboscelidia novoguineana Kimsey 1988: 74. Holotype male; Papua New Guinea, East Highlands, Aiyura (BPBM).

#### Material studied.

Papua New Guinea: Mt. Suckling (1 male, CNC); Ivimka Res. Station, Lakekamu Basin (3 males, BME); 5 males were seen, including the holotype.

#### Diagnosis.

As discussed under *Loboscelidia nigricephala*, *Loboscelidia novoguineana* is one of five species with a wide flattened frontal projection. It can be distinguished from these species by the partial notauli, scrobal sulcus indicated by a scrobal pit or several pits, and the fore, mid and hindtibial flanges present and narrower than the respective tibiae. This is the only *Loboscelidia* species described from New Guinea but there surely must be more.

### Loboscelidia ora

Kimsey

http://species-id.net/wiki/Loboscelidia_ora

Figure 18

Loboscelidia ora Kimsey 1988: 73. Holotype male; Australia: Queensland, Bingil Bay (ANIC).

#### Material studied.

Australia: Queensland: Cape Tribulation (1 male, CNC); Paluma (2 males, CNC, BME); Lacey's Creek, Mission Beach (1 male, CNC); 5 males were examined, including the holotype.

#### Diagnosis.

*Loboscelidia ora* can be distinguished from the other *Loboscelidia* species with an apically broad, flattened frontal projection (Fig. 18) by the nearly straight medial vein, cu-a longer than R, Rs more than twice as long as R, A as long or longer than Cu+M, and no notauli.

### Loboscelidia parva

Maa & Yoshimoto

http://species-id.net/wiki/Loboscelidia_parva

Loboscelidia parva Maa and Yoshimoto 1961: 545. Holotype male; New Britain: Vunabakan, 10 km e Keravat (BPBM).

#### Material studied.

New Britain; only the holotype was seen.

#### Diagnosis.

As discussed under *Loboscelidia cervix* and *Loboscelidia ora*, *Loboscelidia parva* is another of the species with a wide, broadly flattened frontal projection. *Loboscelidia parva* can be distinguished from these species by the arched medial vein, scape 3× as long as broad, partial notauli, pronotum broader than long, and foretibia without a flange.

### Loboscelidia pasohana

Kimsey

http://species-id.net/wiki/Loboscelidia_pasohana

Loboscelidia pasohana Kimsey 1988: 75. Holotype male; Malaysia: Negri Sembilan, Pasoh Forest Reserve (AEI).

#### Material studied.

Malaysia: Negri Sembilan, Pasho Forest Reserve (1 male, AEI); Sarawak: Gunung Mulu NP (4 males, ROM, BME); Sabah: Mt. Kinabalu (2 males, BMNH); NP, Liwagu Rv. Tr. (1 male, CNC); Thailand: Petchaburi, Kaeng Krachan NP (4 males, QSBC); Chiang Mai: Doi Phahompok NP, Mae Fang Hotspring (1 male, QSBC); 12 specimens were seen including the type series.

#### Diagnosis.

This a member of the large group of species with a rectangular frontal projection, submedially curved medial vein, complete scrobal sulcus and complete notauli. *Loboscelidia pasohana* can be distinguished from the rest by the following combination of features: Rs nearly as long as R, cu-a half as long as R, flagellomeres I and II less than twice as long as broad, and fore, mid and hindtibial flanges 1.3–1.7× as wide as tubular part of respective tibiae.

### Loboscelidia pecki sp. n.

urn:lsid:zoobank.org:act:1E7C3500-EE85-481C-AFA6-1DCB27E97A33

http://species-id.net/wiki/Loboscelidia_pecki

Figures 2 14 33

#### Type material. 

Holotype male: Viet Nam: Tuyen Quang Prov., 360 m, Na Hang Reserve, 16–20 May 1997, FIT, S. B. Peck, 97-10 (CNC).

#### Diagnosis.

This species is characterized by the absence of the cu-a vein and having a submedially curved medial vein, characters shared with *Loboscelidia cinnamonea*. It can be distinguished from *Loboscelidia cinnamonea* by Rs 3× as long or longer than R, scape 3× or shorter as long as broad, scrobal sulcus present and the fore and midtibiae without flanges.

#### Male description.

Body (Fig. 2) length 2 mm; forewing length 2.5 mm. Head: length 2× height in side view (Fig. 14); eye asetose; frontal projection rectangular in front view; frons smooth, not microstriate; vertex without transverse fovea, posterior expansion strongly curved in profile; frons with ridge extending from vertex along inner eye margin; gena without scale-like setae; scape striate, length 2.9× breadth; flagellomere I length 2.2× breadth; flagellomere II length 2× breadth; flagellomere XI length 4× breadth. Mesosoma: pronotal length 0.9× breadth, with lateral fold separating dorsal from lateral surface, about as wide as head in dorsal view; scutum with notauli reaching posterior margin; scutellum with fine dense striae laterally; metanotum with three medial ridges enclosing roughened medial area, smooth laterally, 0.4-0.5× as long as scutellum; mesopleuron with scrobal sulcus; propodeum without transverse dorsal carina; legs (Fig. 2) smooth, polished; forefemoral flange 0.6 x femur length, flange maximum width 0.8× width of tubular part of femur; foretibial flange 0.8× tibia length, flange maximum width 0.7 x width of tubular part of tibia; midfemoral flange 0.7× femur length, flange maximum width 0.7× width of tubular part of femur; midtibial flange 0.9× femur length, flange maximum width 0.7× width of tubular part of tibia; hindfemoral flange 0.8× femur length, flange maximum width 0.7× width of tubular part of femur; hindtibial flange equal to femur length, flange maximum width 1.3× width of tubular part of tibia; hindtibia with two longitudinal carinae on posterior margin; hindcoxa without longitudinal carina on inner medial surface; forewing (Fig. 33) R1 length as long as R; cu-a length absent; Rs length 3.2× R length; Cu+M length 0.8× A length; medial vein present, submedially curved. Color: Body dark brown; wing membrane brown-tinted, paler along vein remnants, veins brown.

#### Etymology.

The species is named after the collector, Stuart Peck.

### Loboscelidia philippinensis

Fouts

http://species-id.net/wiki/Loboscelidia_philippinensis

Loboscelidia philippinensis Fouts, 1922: 623. Syntype males (not females); Philippines: Mindanao, Iligan (USNM).

#### Material studied.

Philippines: Mindanao (3 males, USNM, BME); the 2 syntypes were also seen.

#### Diagnosis.

*Loboscelidia philippinensis* is one of the group of species characterized by having a submedially curved medial vein, rectangular frontal projection, no scrobal sulcus, and cu-a vein present. It can be distinguished from the rest of the group by the short, broad head in side view (1.2-1.4× as long as high), flagellomere I is more than twice as long as broad and longer than flagellomere II, partial notauli, metanotum half as long or longer than scutellum, A shorter than Cu+M, and hindtibial flange as long as tibia and twice as wide as tubular part of tibia.

### Loboscelidia reducta

Maa & Yoshimoto

http://species-id.net/wiki/Loboscelidia_reducta

Figures 15 34 45

Loboscelidia reducta Maa and Yoshimoto 1961: 537. Holotype male; Viet Nam: Dai Lanh, n Nha Trang (BPBM).

#### Material studied.

Dai Lanh, Nha Trang (1 male, BPBM); Thailand: Loei: Phu Kradueng NP (3 males, QSBC, BME); Phetchabun: Nam Nao NP (4 males, QSBC, BME); Prachuab Khiri Khan: Khao Sam Roi Yot NP, Laem Sala Beach (2 males, QSBC); Khonkaen: Nam Pong NP (1 male, QSBC); Sakon Nakon, Phu Phan NP (2 males, QSBC, BME), Mae Hong Son: Namtok Mae Surin NP (3 males, QSBC, BME); Chiang Mai: Huai Nam Dang NP (1 male, BME); Kanchanaburi: Khuean Srinagarindra NP, Tha Thung-na/Chong Kraborg (1 male, QSBC); 22 specimens were seen including the holotype.

#### Diagnosis.

*Loboscelidia reducta* is one of the species, including *Loboscelidia incompleta*, *Loboscelidia bakeri*, *Loboscelidia fulgens* and *Loboscelidia ganxiensis*, that have a rectangular frontal projection (Fig. 15), complete notauli, greatly reduced or absent cu-a vein and no medial vein (Fig. 34). It can be distinguished from them by the absence of fore, mid and hindtibial flanges (Fig. 45). This species bears a superficial resemblance to species of *Rhadinoscelidia*.

### Loboscelidia rufa

Fouts

http://species-id.net/wiki/Loboscelidia_rufa

Loboscelidia rufa Fouts 1925: 517. Syntype males; Philippines: Sibuyan (USNM).

#### Material studied.

Philippines: Misamis Or., Mt. Empagatao (1 male, BPBM); Sibuyan (2 males, USNM); Three specimens were seen including the syntypes.

#### Diagnosis.

This is another species in the group with complete notauli, scrobal sulcus and rectangular frontal projection. *Loboscelidia rufa* can be separated from other members of the group by the combination of the hindtibial flange nearly twice as wide as the tubular part of the respective tibiae (shared with *Loboscelidia kafae*), flagellomeres I and II twice as long as broad or longer, and midtibial flange as long and as wide as the tubular part of the tibia.

### Loboscelidia rufescens

Westwood

http://species-id.net/wiki/Loboscelidia_rufescens

Loboscelidia rufescens Westwood 1874: 172. Syntype males (not females); “Sul” (Sula) Isl. (OUMNH).

#### Material studied.

Indonesia: Sula Island, Malaysia: Sarawak; only the 2 syntype of *Loboscelidia rufescens* Westwood were seen.

#### Diagnosis.

*Loboscelidia rufescens* is another of the species characterized by having a curved medial vein, rectangular frontal projection and no scrobal sulcus, as discussed under *Loboscelidia philippinensis*. In this group *Loboscelidia rufescens* differs from *Loboscelidia nasiformis* and *Loboscelidia cinnamonea* in having cu-a well-developed and half as long as R. It can be separated from *Loboscelidia philippinensis*, and *Loboscelidia levigata* by the combination of the scape and flagellomere XI less than twice as long as broad, flagellomeres I and II less than 1.7× as long as broad and hindtibial flange less than 0.7× as wide as tubular part of tibia.

### Loboscelidia sarawakensis

Kimsey

http://species-id.net/wiki/Loboscelidia_sarawakensis

Loboscelidia sarawakensis Kimsey 1988: 75. Holotype male; Sarawak, 4^th^ div., Gn. Lulu (BMNH).

#### Material studied.

Malaysia: Sarawak: Gunung Mulu NP (3 males, ROM, BME); Mentawai Range (1 male, ROM); 4^th^ div., Gunung Lulu (1 male, BMNH); 5 males were seen including the holotype.

#### Diagnosis.

As discussed under *Loboscelidia castanea* and *Loboscelidia collaris*, *Loboscelidia sarawakensis* is one of seven species with a triangular frontal projection, complete scrobal sulcus and complete notauli. *Loboscelidia sarawakensis* can be separated from other members of the group by the combination of scape less than 2.5× as long as broad, flagellomeres I and II twice as long as broad, flagellomere XI 3.3× as long as broad, metanotum 0.3× as long as scutellum, and fore, mid and hindtibial flanges as long as and at least as wide as tubular part of respective tibiae.

### Loboscelidia scutellata

Fouts

http://species-id.net/wiki/Loboscelidia_scutellata

Loboscelidia scutellata Fouts 1922: 628. Syntype males (not females); Philippines: Mindanao, Basilan, Surigao (USNM).

#### Material studied.

Only the 2 syntypes were seen.

#### Diagnosis.

*Loboscelidia scutellata* is another of the species with a complete scrobal sulcus and notauli, and a triangular frontal projection. Characteristics that separate this species from the rest include the scape striate and 2.5–2.7× as long as broad, flagellomeres I and II twice as long as broad, flagellomere XI 2.4× as long as broad, fore and midfemoral flanges less than half as long as femora, hindtibial flange as long as tibia and 0.6× as wide as tubular part of tibia.

### Loboscelidia sinensis

Kimsey

http://species-id.net/wiki/Loboscelidia_sinensis

Loboscelidia sinensis Kimsey 1988: 76. Holotype male; China: Hainan Island, Tien Fong Mts. (ZFCL).

#### Material studied.

Only the holotype was seen.

#### Diagnosis.

This is the last of the species group discussed under *Loboscelidia scutellata*. *Loboscelidia sinensis* can be distinguished from the rest by the short R1 vein (0.2× as long as R), A as long as Cu+M, scape twice as long as broad, flagellomeres I and II 2.5× as long as broad, and metanotum 0.3× as long as scutellum.

### Loboscelidia sisik sp. n.

urn:lsid:zoobank.org:act:10FCFF3D-8DE8-4511-8671-85B5934C1A1D

http://species-id.net/wiki/Loboscelidia_sisik

Figures 16 35 46

#### Type material.

Holotype male: Borneo, W. Kalimantan, Gunung Palung Nat. Pk., 15 June-15 Aug. 1991, Darling, Ubaidillah (Rosichon), Sutrisno, 11S 910131 (MBBJ).

Paratype: 1 male, same data as holotype, 11S 910125 (BME).

#### Diagnosis.

This species has a number of distinctive features that in combination will distinguish it from other *Loboscelidia*, including the scale-like setae on the head and legs and the scrobal sulcus reduced to a series of foveae.

#### Male description.

Body length 3–4 mm; forewing length 3.5–4.5 mm. Head (Fig. 16): length 1.7× height in side view; eye asetose; frontal projection triangular in front view; frons smooth, not microstriate; vertex without transverse fovea, posterior expansion strongly convex in profile; frons with ridge extending from vertex along inner eye margin; gena without scale-like setae; scape smooth, not striate, length 1.8 breadth; flagellomere I length 1.8× breadth; flagellomere II length 2.8× breadth; flagellomere XI length 3.5× breadth. Mesosoma: pronotal length 0.8× breadth, with lateral carina, as wide as head in dorsal view; scutum with notauli reaching posterior margin; scutellum with scattered large punctures and fine dense striae posteriorly; metanotum medially finely, densely striate/punctate impunctate laterally. 0.3× as long as scutellum; mesopleuron with scrobal sulcus consisting of 3–4 large pits or foveae; propodeum without transverse dorsal carina; legs (Fig. 46) smooth, polished; forefemoral flange 0.7 x femur length, flange maximum width 0.6× width of tubular part of femur; foretibial flange 0.4× tibial length, flange maximum width 0.4 x width of tubular part of tibia; midfemoral flange 0.5× femur length, flange maximum width as wide as tubular part of femur; midtibial flange absent; hindfemoral flange 0.9× femur length, flange maximum width as wide as tubular part of femur; hindtibial flange 0.9× tibial length, flange maximum width 0.6× width of tubular part of tibia; hindtibia with two longitudinal carinae on posterior margin; hindcoxa with longitudinal carina on inner medial surface; forewing (Fig. 35) R1 length 0.5× R length; cu-a length 0.7× R length; Rs length 2.3× R length; Cu+M as long as A; medial vein submedially curved. Color: body dark brown; wing membrane brown-tinted, darker along vein remnants.

#### Female.

Unknown.

#### Etymology.

The species name is Indonesian for scale, referring to the scale-like setae on the head and legs (noun).

### Loboscelidia striolata

Yao, Liu & Xu

http://species-id.net/wiki/Loboscelidia_striolata

Loboscelidia striolata Yao, Liu & Xu, 2010: 528. Holotype male; China: Guangdong Prov., Nanking National Nature Reserve (SCAC).

#### Material studied.

None.

#### Diagnosis.

*Loboscelidia striolata* may very well be part of the species group discussed under *Loboscelidia castanea* and *Loboscelidia collaris*, characterized by a triangular frontal projection, complete scrobal sulcus and complete notauli. However, the published description and images do not show the mesopleuron clearly enough to determine whether the scrobal sulcus is present or not. If it does have a scrobal sulcus then *Loboscelidia striolata* may be synonymous with *Loboscelidia sinensis*. Both *Loboscelidia striolata* and *Loboscelidia sinensis* share similar head, wing vein, flagellar and leg flange dimensions. They appear to differ in the dimensions of the scape, which 3× as long as broad in *Loboscelidia striolata* and twice as long as broad in *Loboscelidia sinensis* and possibly in the presence of the scrobal sulcus in *Loboscelidia sinensis*.

#### Female.

Unknown.

## Supplementary Material

XML Treatment for Loboscelidia antennata

XML Treatment for Loboscelidia asiana

XML Treatment for Loboscelidia atra

XML Treatment for Loboscelidia australis

XML Treatment for Loboscelidia bakeri

XML Treatment for Loboscelidia brunnea

XML Treatment for Loboscelidia castanea

XML Treatment for Loboscelidia cervix

XML Treatment for Loboscelidia cinnamonea

XML Treatment for Loboscelidia collaris

XML Treatment for Loboscelidia defecta

XML Treatment for Loboscelidia fulgens

XML Treatment for Loboscelidia fulva

XML Treatment for Loboscelidia guangxiensis

XML Treatment for Loboscelidia halimunensis

XML Treatment for Loboscelidia incompleta

XML Treatment for Loboscelidia indica

XML Treatment for Loboscelidia inermis

XML Treatment for Loboscelidia kafae

XML Treatment for Loboscelidia laminata

XML Treatment for Loboscelidia laotiana

XML Treatment for Loboscelidia levigata

XML Treatment for Loboscelidia maai

XML Treatment for Loboscelidia maculata

XML Treatment for Loboscelidia maculipennis

XML Treatment for Loboscelidia meifungae

XML Treatment for Loboscelidia nasiformis

XML Treatment for Loboscelidia nigra

XML Treatment for Loboscelidia nigricephala

XML Treatment for Loboscelidia nigricornis

XML Treatment for Loboscelidia nitidula

XML Treatment for Loboscelidia nixoni

XML Treatment for Loboscelidia novoguineana

XML Treatment for Loboscelidia ora

XML Treatment for Loboscelidia parva

XML Treatment for Loboscelidia pasohana

XML Treatment for Loboscelidia pecki

XML Treatment for Loboscelidia philippinensis

XML Treatment for Loboscelidia reducta

XML Treatment for Loboscelidia rufa

XML Treatment for Loboscelidia rufescens

XML Treatment for Loboscelidia sarawakensis

XML Treatment for Loboscelidia scutellata

XML Treatment for Loboscelidia sinensis

XML Treatment for Loboscelidia sisik

XML Treatment for Loboscelidia striolata
